# Video-based messages to reduce COVID-19 vaccine hesitancy and nudge vaccination intentions

**DOI:** 10.1371/journal.pone.0265736

**Published:** 2022-04-06

**Authors:** Ulrich T. Jensen, Stephanie Ayers, Alexis M. Koskan

**Affiliations:** 1 School of Public Affairs, Arizona State University, Phoenix, Arizona, United States of America; 2 Crown Prince Frederik Center for Public Leadership, Aarhus University, Aarhus, Denmark; 3 School of Social Work, Arizona State University, Phoenix, Arizona, United States of America; 4 Southwest Interdisciplinary Research Center, Arizona State University, Phoenix, Arizona, United States of America; 5 College of Health Solutions, Arizona State University, Phoenix, Arizona, United States of America; UCLA Fielding School of Public Health, UNITED STATES

## Abstract

Vaccines are highly effective for curbing the spread of SARS-CoV-2 (COVID-19). Yet, millions of Americans remain hesitant about getting vaccinated, jeopardizing our ability to end the COVID-19 pandemic by fueling the spread and development of new variants. We show that brief video-based messages of encouragement addressing specific COVID-19 vaccine concerns increase vaccination intentions, and that vaccination intentions, in turn, are predictive of future vaccine uptake. Results from our online experiment reveal that willingness to get vaccinated is driven by messages that increase confidence in COVID-19 vaccines and perceived behavioral control to get vaccinated. Importantly, messages were particularly effective among more skeptical populations including people who identify as politically conservative or moderate and those who express low trust in government institutions. Our findings corroborate the real-world behavioral significance of vaccination intentions, and devise how even short, scalable online messages can provide governments and health authorities an inexpensive, yet effective tool for increasing intentions to vaccinate against COVID-19 among populations most reluctant to get them.

## Introduction

Vaccines are highly effective for curbing the spread of SARS-CoV-2 (COVID-19), reducing hospitalizations, and saving lives [1, 2]. Yet, a large percentage of Americans remains hesitant, with millions still not vaccinated against this virus [3, 4]. Vaccine supply is widely reported to exceed demand, with conservative, rural, and younger populations expressing greater COVID-19 vaccine hesitancy [5, 6]. This is particularly concerning as the virus mutates to create new, more transmissible variants like the Delta and Omicron strains currently ravaging communities across the US [7]. In addition, scientists have warned that people may not follow through on their expressed intentions to get vaccinated [8]. Forecasts and preliminary trends from other countries, such as Denmark, document the power of high vaccination rates for effectively eliminating COVID-19 outbreaks [9], yet the fully vaccinated percentage of the US population still falls short of those targets (~64% as of February 4, 2022; [4]). Given the pandemic’s enormous human and economic costs [10], it is incumbent on researchers to identify evidence-based strategies that can be easily scaled, offered at a low cost, and rapidly deployed to reduce vaccine hesitancy and increase willingness to get vaccinated against COVID-19, especially among the parts of the current population most skeptical of getting vaccinated.

Past research emphasizes message framing as an important part of identifying the type of messages that most resonate and influence audiences’ intentions to vaccinate, for instance, by couching messages in terms of future gains (benefit of enacting a behavior) rather than future losses (negative outcome resulting from lack of enacting a behavior) for promoting prevention behaviors [11]. Recent studies into willingness to vaccinate against COVID-19 suggest that both gain-framed messages emphasizing vaccinating for self-protection (individual-centered messages) [12], and messages focusing on the community benefit of vaccination (community-centered messages) [13] can be effective at enhancing individuals’ willingness to vaccinate. Combining gain-framed individual- and community-centered messages might even have synergistic effects, creating the greatest impact on intentions to vaccinate against COVID-19 [14].

Research at the crossroads between behavioral science and public health has also pointed to the effectiveness of nudges for increasing vaccinations [15–19]. For example, recent evidence shows that text message reminders to patients ahead of planned appointments increase influenza vaccine uptake by as much as 6.7% [17]. This follows other studies that showcased the power of providing reminders [18], prompting people to write down appointment details [19], or mailing letters of encouragement [20] for increasing influenza vaccinations. Recent evidence shows that similar approaches can also be effective for COVID-19 vaccine uptake [21] by increasing individuals’ sense of psychological ownership of vaccines [21, 22].

In other studies, COVID-19 research has built on and extended communication strategies shown to be effective at increasing vaccine acceptance for other viruses (e.g., influenza or HPV). As a case in point, Dai et al. [21] followed Milkman et al.’s [17] successful nudge to make vaccination salient and easy by reminding individuals via text messages that a shot was reserved for them. Although text-based reminders and other nudges can play an important role for boosting uptake, less evidence exists on complementary interventions focusing on vaccine hesitancy, especially among more vaccine-skeptical populations. It is, therefore, paramount to continue identifying ways to expand our toolkit of nudges and behavioral interventions that can be used to fight COVID-19 and future health crises.

We heed this call by testing four video-based messages, all designed to reduce vaccine hesitancy and encourage viewers’ willingness to vaccinate against COVID-19. In designing our video-based messages, we drew on the Theory of Planned Behavior; a well-validated framework in vaccine research for understanding the antecedents of vaccine hesitancy [23–25]. Vaccine hesitancy is commonly asserted to be rooted in low confidence in the vaccine, manifesting, for instance, as negative attitudes about the efficacy or safety of the vaccine, lack of perceived behavioral control to overcome barriers to receive the vaccine, and the absence of a sense of social expectation (norm) to inoculate [26]. Applied to COVID-19, we expect individuals who believe that the vaccine is safe and effective and that others want them to vaccinate will be more likely to express willingness to get vaccinated. Similarly, individuals who are confident in their ability to get vaccinated and who find it easy and convenient to get vaccinated will be more likely to express willingness to get vaccinated. Finally, we expect individuals to express less hesitancy and greater willingness to get vaccinated if they believe the vaccine to be efficacious in stopping virus transmission, alleviating the economic impact of the pandemic, and generally safe to get.

## Materials and methods

To test how brief, low-cost online messages can reduce vaccine hesitancy and increase willingness to get vaccinated, we created a two-wave balanced panel of 890 adults living in the United States and embedded a video-based between-subjects randomized experiment as part of the second survey. Of these 890 individuals, 447 were not fully vaccinated at T2 –the time of our experiment–and hence constitute the “treatable” subsample for our main results.

The first survey (T1) was conducted in January/February 2021 with 1,620 total respondents recruited via Amazon’s online labor market platform, Mturk. To ensure high-quality responses, we screened out international respondents based on VPN/VPS use [27] and disguised an attention check as part of a 5-question battery. See S1 Appendix for details on screening protocol and sample representativeness. We used the survey to obtain demographic information (e.g., gender, age, race/ethnicity, education, political ideology) and measures of COVID-19 vaccine perceptions. Such perceptions included baseline measures of vaccination intentions (two-digit continuous sliding scale from 0, completely unwilling, to 10, completely willing), trust in government institutions to provide accurate information on COVID-19 (5-point Likert scale from “completely distrust” to “completely trust”), and vaccine beliefs (5-point Likert scale items; e.g., “I am capable of getting the COVID-19 vaccine.” See S1 Table for all measures).

We conducted the second survey (T2) in May 2021 after COVID-19 vaccinations became widely available for all adults. A total of 890 individuals completed the survey, 447 of whom self-reported not to be fully vaccinated at the time of the survey. Of our initial 1,620 respondents, 112 individuals had already received a COVID-19 vaccine, leaving us with a final pool of 1,471 potential respondents after discarding 37 people for whom the system did not capture their unique identifier. Individuals were invited to participate in our T2 follow-up survey via a personalized email prompt from CloudResearch [28]. 890 responses represent a retention rate of 60.5%, with 447 making up 30.4% of our original sample. Panel attrition analyses are presented in S2 Appendix. We repeated our measurements of vaccine beliefs and vaccination intentions. We also incentivized respondents who reported being vaccinated to share a redacted version of their official CDC vaccination card for validation purposes (see S3 Appendix for details). Sharing of CDC card was not predicted by baseline vaccination intention. Finally, respondents completed two attention checks to gauge whether they were able to remember basic information from the video messages. See S4 Appendix for survey flowcharts.

At T2, prior to being asked to respond to the questions described above, respondents were randomized to view one of five brief videos encouraging them to get vaccinated against COVID-19. Four different treatment videos were created based on the Theory of Planned Behavior [29] and targeted different beliefs and attitudes theorized to be crucial drivers of vaccine hesitancy [30, 31]: (i) attitudes about vaccine safety, (ii) normative beliefs about the subjective, social norm to get vaccinated, (iii) attitudes about vaccine efficacy (response efficacy), and (iv) perceived behavioral control to get a vaccine (self-efficacy). As an example, our “response efficacy” nudge script reads *“… Did you know*? *Vaccines are so effective that the risk of getting infected with COVID-19 is reduced 90% after two doses*. *Vaccines are the number one healthcare resource for stopping the pandemic*, *fully re-opening the economy*, *and getting us back to our pre-COVID “normal”*. *So*, *what are you going to do*?” See S5 Appendix for scripts and recordings of all five videos.

Videos were of similar length (range: 29–32 seconds; 72–73 words) and kept brief to mimic short video ads that could air on TV or online platforms like YouTube. Videos featured the same actor portraying a pharmacist—as this group of healthcare professionals are seen as highly trustworthy among more vaccine-hesitant populations [32]. Video scripts had an identical intro and exit. We only varied the nudge or substantive reason for getting vaccinated. Finally, we designed all scripts to be equally appealing in terms of their use of charismatic communication techniques [33, 34].

The four treatment videos were all compared to an active control condition which described how vaccines work to protect the body. We designed our control script to act as a placebo video in the sense that it delivers a standard motivational prompt and encourages vaccination similar to the sentiment of the treatment videos. (See the full script in S5 Appendix). This is critical to avoid experimenter demand effects [35]. Furthermore, the control script is of the same length, and equally charismatic as the treatment scripts. This is instrumental in order to avoid confounding quantitative and qualitative aspects of our messages. By giving all respondents videos of similar look, feel, and basic message (to vaccinate against COVID-19), but only varying the theoretical and substantive reason used to nudge vaccination behavior, we can isolate the effects of our treatment messages on reducing vaccine hesitancy and increasing willingness to get vaccinated.

Both surveys and the experiment were approved by the Institutional Review Board at Arizona State University. Subjects provided informed consent to participate in the study on the first page of each survey. The experiment, including all protocols and the main analysis plan, were preregistered with the Open Science Framework prior to collection of data (see https://doi.org/10.17605/OSF.IO/EHZAU). As part of the preregistration, the minimum required sample size for linear regression/ANCOVA models was calculated as 297 individuals, based on the assumptions of a moderate to strong autoregressive component with T1 vaccination intentions explaining approximately 50% of the variance in T2 vaccination intentions, five groups, 80% power, an error rate of 5%, and a small substantive effect size of approximately 0.14 (~2% explained variance in T2 vaccination intentions). Our final sample is one and a half times this number providing for well-powered tests of our messages.

To estimate the effect of our video messages, we conducted OLS regression analyses with four indicator variables denoting treatment status, or a single indicator variable when pooling data across treatments. (See S6 Appendix for details on identification strategy). All models control for T1 vaccination intentions. Statistical tests are two-tailed and report statistical significance as 95% confidence intervals.

## Results

Respondents for our main analyses (*n* = 447) were 49.4% men, had an average age of 40.7 years (SD = 11.2), were predominantly white (73.4%), had a college degree (50.1%), lived in urban areas (74.5%), and primarily identified as politically conservative (46.1%; 32.0% identified as politically liberal and 21.9% as moderate). At T1, 49.1% indicated willingness to get vaccinated. At T2 (3 months later), vaccination intentions remained stable, with 50.3% expressing willingness to get vaccinated. The temporal stability in average vaccination intention strongly suggests that our sample comprises vaccine-hesitant individuals who, on average, did not move towards greater vaccine acceptability as a function of time. These numbers also trend well below national polls at the time [36], giving further credence to the argument that this sample constitutes an appropriate setting for testing the effectiveness of our experimental video-based messages.

Importantly, among all respondents who completed the survey (*n* = 890), 68.6% indicated that they would be willing to get vaccinated at T1, tracking close to rates reported in national polls conducted at the same time [36]. Thus, respondents in our panel were no less vaccine-hesitant or no more vaccine-accepting than the general American population. At T2 (3 months later), this 73.0% of our respondents reported willingness to receive a COVID-19 vaccine (repeated measures correlation: 0.81). While none of our respondents had received a COVID-19 vaccine at T1, at T2 57.2% were partially or fully vaccinated. This closely resembles the 61.6% of American adults who had received at least one vaccination shot at the time our survey concluded on May 26, 2021 [37]. At T2, 447 respondents reported not being fully vaccinated, making up the sample for our main analyses. Sample statistics can be found in S1 and S2 Tables and assessment of representativeness in S1 Appendix. Correlation matrix for study variables can be found in S3 Table.

Assignment to an experimental group or the placebo condition was well-balanced across respondent demographic covariates: gender, age, race/ethnicity, education, political ideology, and urban/rural living, (*F*(9, 437) = 1.54, *p* > 0.05). When differentiating between all five experimental groups, we observe a small skewness across groups on participants’ gender. This could be a function of chance, but as a precaution, we adjusted treatment estimates for this and other covariates in secondary analyses. In addition to our final sample of 447 unique individuals (894 observations), 15 individuals failed to recall either the correct gender of the actor (*n* = 14) or the core sentiment to get the vaccine (*n* = 1). Although the number of people failing the attention check is low, we included these individuals in secondary analyses to assess the robustness of our results. (See S4 Table) Another 5 respondents provided incomplete information and were dropped from the main analyses. Attrition due to failed attention checks was not predicted by assignment to treatments (*P* values greater than 0.05 for all treatment indicators), nor by baseline vaccination intention or demographic covariates (*F*(14, 449) = 1.32, *p* > 0.05).

To investigate whether participants expressed greater intentions to get vaccinated following our messages, we conducted OLS regression analyses with four indicator variables denoting treatment status, or a single indicator variable when pooling data across treatments. (See S6 Appendix for details on identification strategy.) In either case, assignment to the placebo condition represents the omitted value, and hence the counterfactual for all treatment effect estimates. All models controlled for T1 vaccination intentions to improve power and measurement precision [38]. See S4 and S5 Tables for extended analyses with adjustment for demographic covariates.

**Fig 1:**
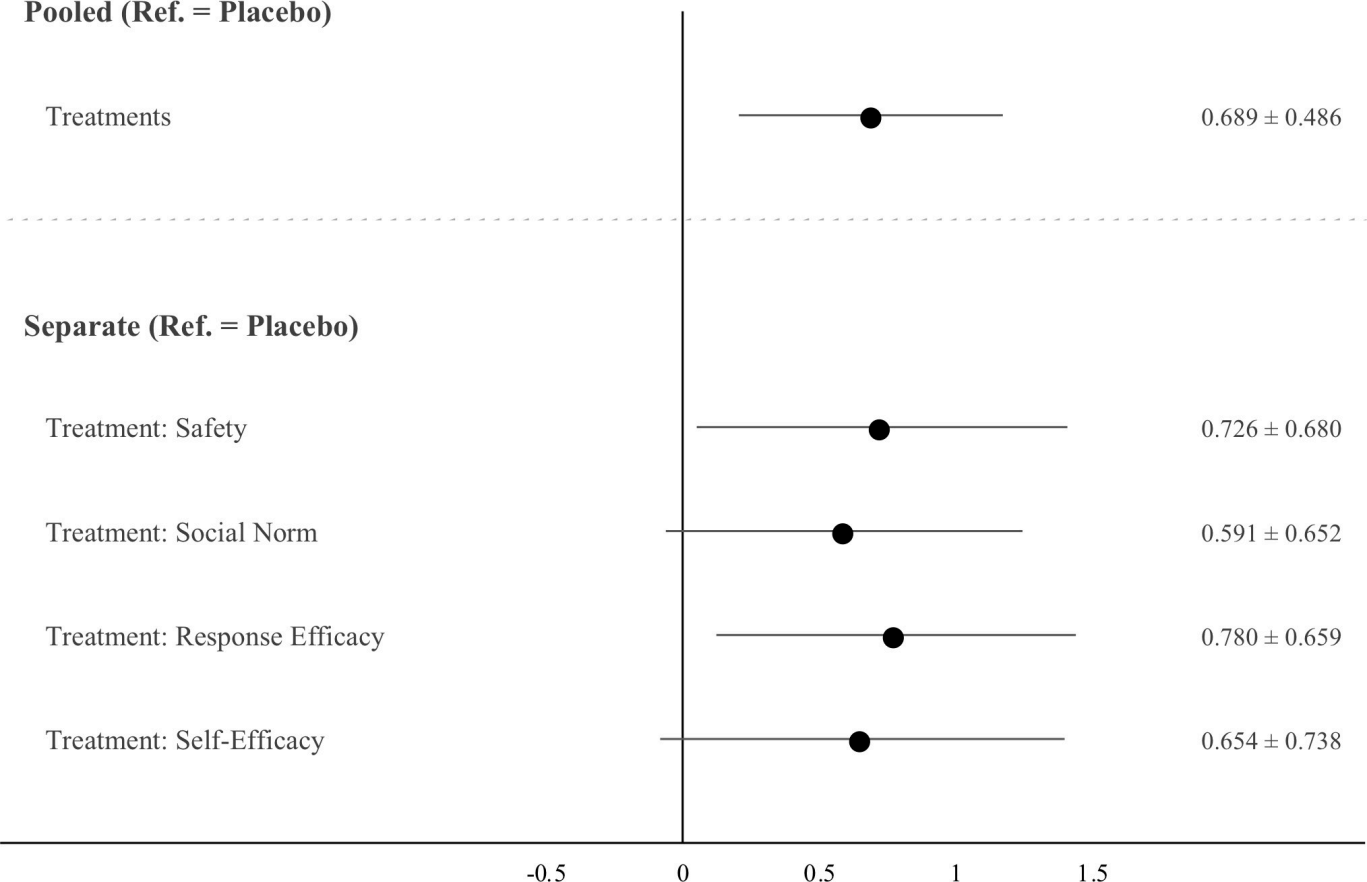
Increase in vaccination intentions after watching treatment videos. N = 447 (N_placebo_ = 113, N_safety_ = 94, N_social norm_ = 79, N_response efficacy_ = 81, and N_self-efficacy_ = 80). Markers represent ATEs as unstandardized regression coefficients based on OLS. ATEs and 95% CIs listed on right-hand side. Estimated using heteroscedastic robust standard errors.

Fig 1 shows average treatment effects of the video messages on unvaccinated respondents as unstandardized regression coefficient estimates with heteroscedastic robust standard errors and 95% confidence intervals. Compared to the placebo video, all four treatment messages increased intentions to get vaccinated (with two messages having *P* values from two-sided tests < 0.05, and two messages having *P* values from two-sided tests < 0.10). A pooled treatment variable produced a statistically significant boost in vaccination intentions (b = 0.69, *p* = 0.006), with a standardized effect of 0.187. Standardized betas show effect sizes of individual messages that track closely with or exceed our preregistered expectations: ß_safety_ = 0.197, ß_social norm_ = 0.160, ß_response efficacy_ = 0.211, ß_self-efficacy_ = 0.177.

Scientists have warned that people may not follow through on their intentions, putting into question the behavioral implications of our findings for vaccination uptake [8, 17]. To help demonstrate their real-world significance for vaccination behavior, and to offer a back-of-the-envelope calculation of their effects, we leveraged our repeated measures design to predict both self-reported vaccination status as well as objectively verified vaccination status (T2) as a function of past vaccination intentions (T1) among our full set of respondents with complete information (*n* = 843). In other words, we assessed whether individuals who expressed greater willingness to get vaccinated in January/February 2021 were more likely to be vaccinated by May 2021.

Descriptive results demonstrated a clear connection between vaccine intentions at T1 and uptake by T2. Among individuals expressing hesitancy at T1 (i.e., values 5 or lower on 0–10 scale), only 13.2% reported being vaccinated by May 2021. For respondents reporting willingness to get vaccinated at T1 (values higher than 5 on our scale), 75.0% reported being vaccinated at T2.

More sophisticated OLS regressions with or without an extensive set of demographic and vaccine belief controls corroborated this pattern (see S6 Table for full estimation results). The estimated regression coefficient of T1 vaccination intention on self-reported T2 vaccination status was 0.083, suggesting that an individual completely willing to get vaccinated at T1 has an 83% higher likelihood of being vaccinated by May compared to an individual being completely unwilling to get vaccinated. Relying solely on objectively verified vaccination status based on shared redacted CDC vaccination cards, the corresponding likelihood was estimated at 33%. However, this estimate is very conservative as we trade-off verification for false negatives (i.e., people who are vaccinated but unwilling to share their card are classified as unvaccinated by assuming a social desirability factor of 1).

For the purpose of a back-of-the-envelope calculation of the effects of our treatment messages for vaccination behavior, we used the pooled ATE of a 0.69-point increase in vaccine intentions. Using the most liberal estimate noted above, a one-point increase in vaccine intentions leads to an 8.3% increase in vaccine uptake, implying that our treatments could induce an approximate increase in future uptake of 5.7%. The most conservative estimate puts this at a 2.3% increase. Even by this conservative estimate, with millions of Americans still unvaccinated, our messages video-based messaging could potentially boost inoculation numbers by tens of thousands if equally effective in the field and scaled as part of national vaccination campaigns.

**Fig 2:**
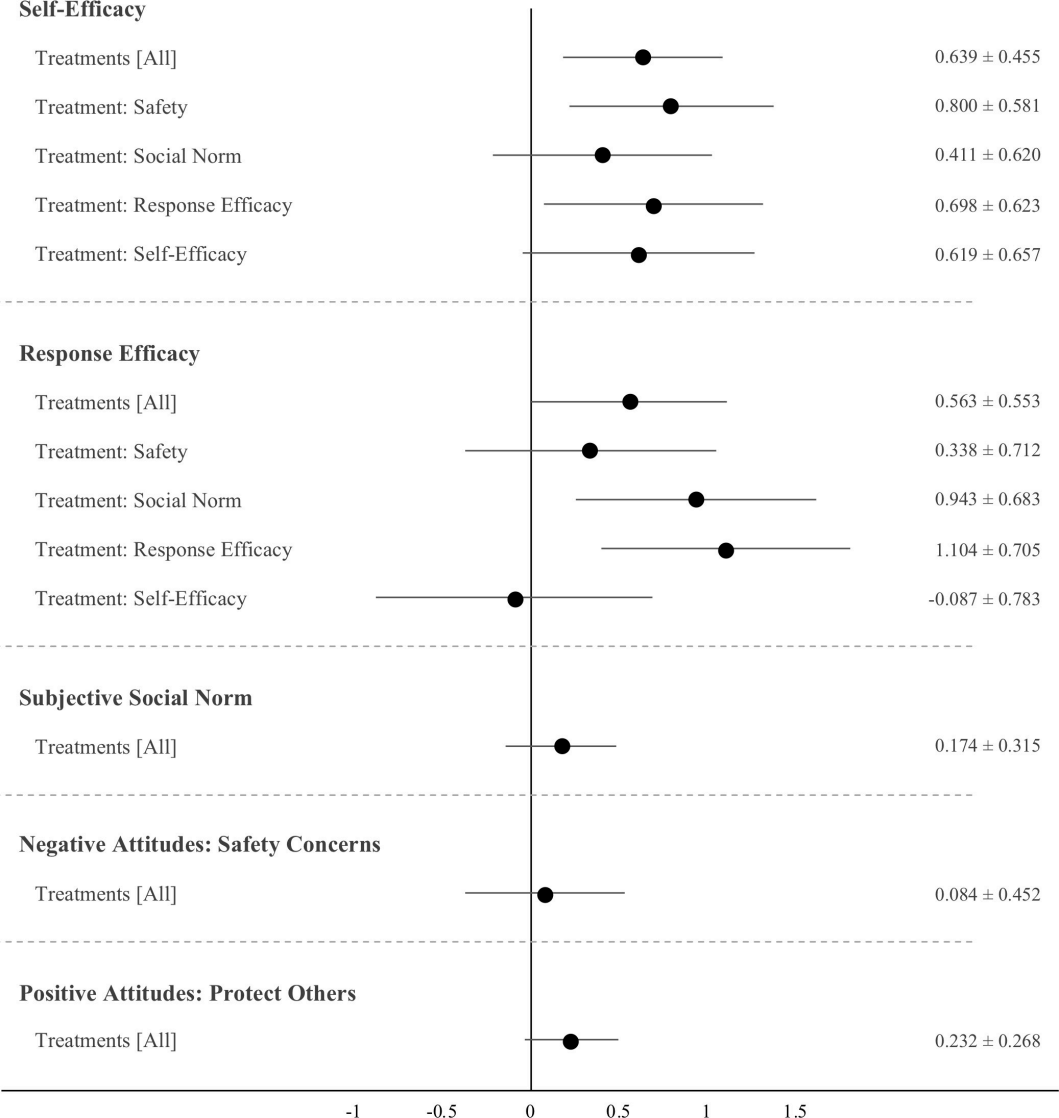
Increase in psychological drivers of vaccine hesitancy after watching treatment videos. N = 447. Markers represent ATEs as unstandardized regression coefficients based on OLS. ATEs and 95% CIs listed on right-hand side. Estimated using heteroscedastic robust standard errors. No systematic effects of treatments were observed for DVs: Subjective social norm, safety concerns or protect others.

In post-hoc analyses, Fig 2 reports estimated ATEs of the treatment messages on five proximate psychological beliefs presumed to drive hesitancy [39]. Several treatment videos increased individuals’ perceived behavioral control (self-efficacy) and belief in vaccines as an effective response for combatting the pandemic. No effects are found for the perceived social norm to get vaccinated nor on positive (desire to protect others) or negative (safety concerns) attitudes. See S7 and S8 Tables for full results.

Since treatment messages offer exogenous variation in the psychological concepts of self-efficacy and response efficacy, they can be considered experimentally randomized instrumental variables [40] and used in two-stage least-squares estimation to identify the causal effect of self-efficacy and response efficacy on vaccination intentions. Table 1 reports first-stage and second-stage results from our econometric analyses (see S6 Appendix for details on estimation and S9 Table for full results). The second stage results indicate that self-efficacy and response efficacy both have positive effects on vaccination intention, suggesting these make up important psychological mechanisms in explaining the effectiveness of our messages on vaccination intentions.

**Table 1:** Response efficacy and self-efficacy as psychological mechanisms driving the effect of video messages on vaccination intentions.

Stage:	First	Second	First	Second
Dependent Variable (T2):	Self-Efficacy	Vaccine Intention	Response Efficacy	Vaccine Intention
Experimental Messages *(Ref*. *= Placebo)*				
Treatment: Response Efficacy			1.16***	
			(2.91)	
Treatment: Self-Efficacy	0.998**			
	(2.51)			
Self-Efficacy (T2)		0.732*		
		(1.69)		
Response Efficacy (T2)				0.570*
				(1.83)
Constant	12.76***	-8.34	7.38***	-3.58
	(11.45)	(-1.44)	(7.71)	(-1.30)
Controls	YES	YES	YES	YES
Observations	193	193	194	194
R-squared	0.09	0.48	0.48	0.69

Instrumental variables regression using video treatments as experimentally randomized instrumental variables. Regression coefficients report first and second stage result adjusted for respondent characteristics, including: gender, age, race/ethnicity, education, political ideological orientation, rural/urban living and baseline (T1) vaccination intentions.

*** p<0.01

** p<0.05

* p<0.1. Heteroscedastic robust t-statistic in parentheses.

**Fig 3:**
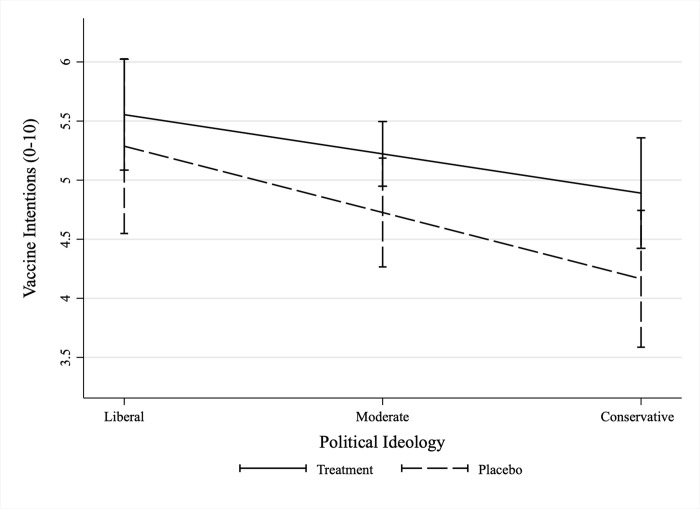
Heterogenous treatment effect on vaccination intentions by political ideology. N = 447. Effect estimates are LATE. Unstandardized regression coefficients based on OLS with 95% CIs. Estimated using heteroscedastic robust standard errors.

For exploratory purposes, we assessed potential heterogeneity in the receptivity to our messages along individuals’ political ideology and trust in government institutions. That is, we explored the question of whether ATEs are a result of treatment messages preaching to the choir (i.e., reinforcing the motivation among more vaccine accepting individuals who are not yet vaccinated), or if our messages work by persuading more vaccine-hesitant and skeptical groups of the population? Preferences for public health measures, including physical distancing, mask wearing, and vaccination differ along political ideology [41–43], and the gap between vaccination rates in blue and red states have continued to grow [5]. We interacted the pooled treatment indicator with self-reported political ideology (conservative, moderate, or liberal, see S10 Table), and plotted the marginal effects of the treatment messages for each subgroup in Fig 3. Fig 3 indicates that messages boosted vaccination intentions among conservatives (b = 0.73, *p* = 0.061) and among moderates (b = 0.50, *p* = 0.074), but not among liberals who already had intentions to vaccinate (b = 0.27, *p* = 0.531).

**Fig 4:**
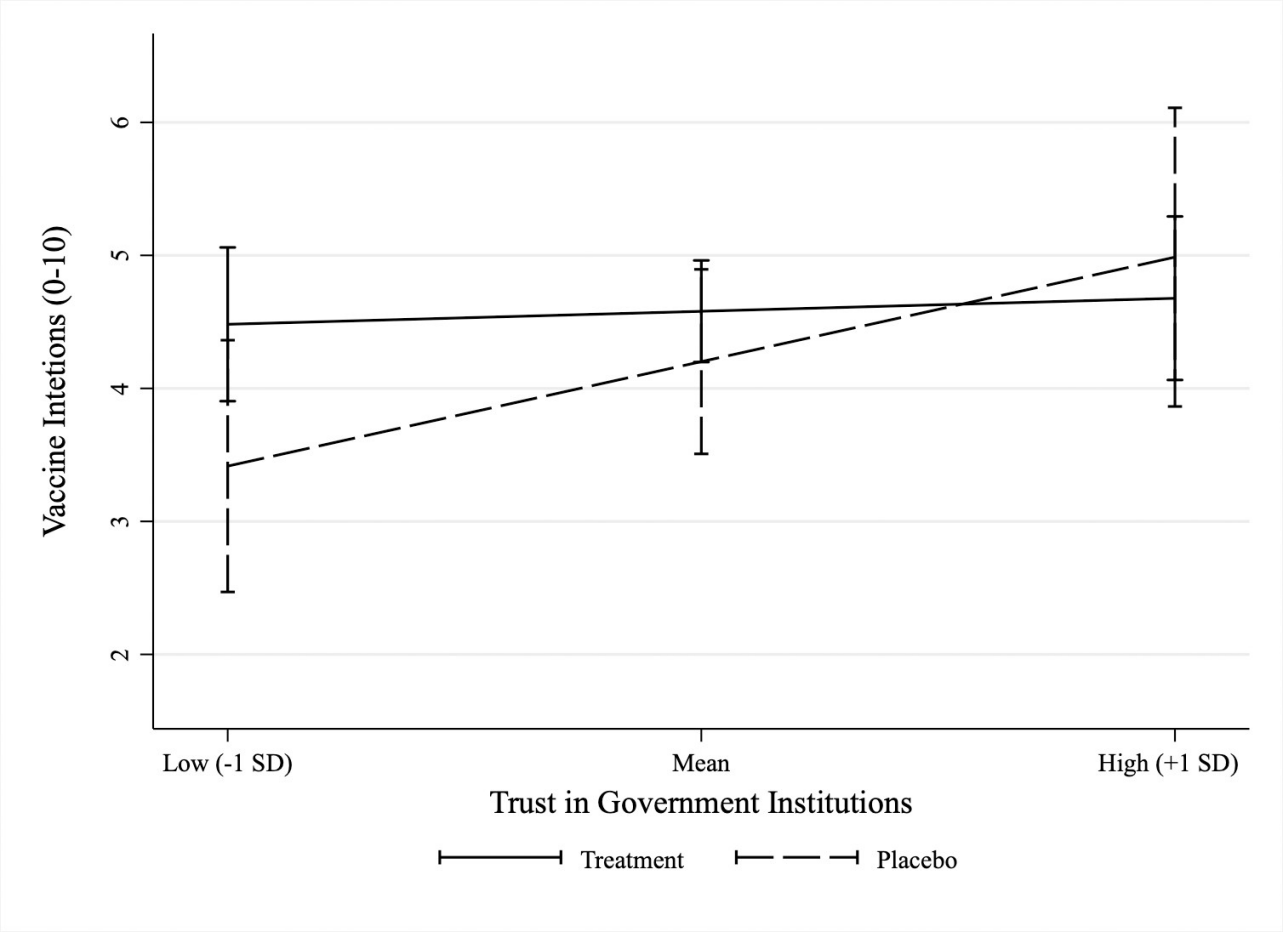
Heterogenous treatment effect on vaccination intentions by trust in government institutions. N = 228. (N_placebo_ = 56, N_safety_ = 48, N_social norm_ = 38, N_response efficacy_ = 47, and N_self-efficacy_ = 39). Effect estimates are LATE. Unstandardized regression coefficients based on with 95% CIs. Estimated using heteroscedastic robust standard errors.

Trust in public leaders and institutions are commonly highlighted as a critical precondition for citizen compliance with public health guidance [44, 45], such as the recommendation to get vaccinated against COVID-19. Using two 5-point Likert scaled items capturing respondents self-reported trust in the US government and the US Coronavirus Taskforce to provide accurate and reliable information on COVID-19 (measured at T1, baseline), we generated an index (mean = 5.6, SD = 2.2, range 2–10) and interacted the index with the pooled treatment indicator (see S11 Table). Fig 4 plots the marginal effects of our treatment messages for respondents with low (-1 SD below mean), average (mean), and high (+1 SD above mean) levels of trust in government institutions. Fig 4 indicates that messages boosted vaccination intentions among individuals expressing low trust in government institutions (b = 1.09, *p* = 0.052), but not among more trusting individuals (b = 0.39, *p* = 0.347 and b = -0.31, *p* = 0.619, respectively). While exploratory, these results are encouraging as they strongly suggest that our treatment scripts can be used to increase the willingness to vaccinate against COVID-19 among current groups most reluctant to get vaccinated.

## Discussion and conclusion

In this study, we showed that brief, theoretically-informed online videos can decrease vaccine hesitancy and increase willingness to vaccinate against COVID-19. In general, vaccine hesitancy is influenced by (i) confidence (negative or positive attitudes such as trust) in the vaccine, (ii) complacency (perceived need to vaccinate), (ii) constraints (accessibility, availability, and psychological barriers), (iv) calculation (need for finding and processing information about the vaccine), and (v) collective responsibility (the desire to vaccinate to protect others) [26].

Our results corroborated the importance of some of these psychological drivers for reducing hesitancy towards COVID-19 vaccines. Our messages reduced hesitancy and increased willingness to vaccinate through two specific pathways. The first is by elevating confidence in vaccines. This included targeting the perceptions of COVID-19 vaccine efficacy in changing the pandemic’s social, economic and public health trajectories. The second pathway worked by removing psychological constraints through increasing perceived behavioral control or self-confidence to get vaccinated. If people find it easy, convenient, and within their abilities to get vaccinated, they are more likely to pursue this behavior. Our scripts devise ways to target these beliefs as part of specific campaign messages from trusted, non-political sources such as pharmacists [46–48]. They also encourage further exploration of tools for elevating unvaccinated individuals’ confidence in the efficacy and safety of vaccines as well as perceived behavioral control to get vaccinated.

Importantly, we not only show that messages can increase vaccination intentions, but that such intentions strongly predict uptake of COVID-19 vaccines. Past critiques have warned that people may not follow through on their intentions [8, 17], putting into question the validity of frameworks such as the Theory of Planned Behavior [49] and existing studies focusing on intentions to vaccinate against COVID-19 as their ultimate outcome [e.g., 50–54]. Using both self-reported vaccination status, and objectively verified vaccination status based on redacted CDC vaccination cards, our results corroborate the behavioral significance of expressed willingness to get vaccinated on future vaccination behavior.

Our study also adds to existing work in important ways. Research on health communication and message framing has highlighted the importance of emphasizing gained-framed messaging and emphasizing prosocial aspects of vaccinations and their public health purpose [e.g., 55]. Recent work in the context of COVID-19 echoes this sentiment, concluding that “helping loved ones” was the most effective appeal for increasing willingness to vaccinate against COVID-19 in randomized trials with 20,000 people [56]. While prosocial communication arguably is essential in the fight against COVID-19, our results suggest that appeals targeting vaccine confidence and perceived behavioral control represent important additional tools that can complement emotion-based appeals to a moral obligation and collective responsibility.

Despite a strong emphasis on leveraging normative beliefs to increase vaccinations [53, 54], this message was the least effective appeal for generating a main effect on vaccination intentions in our study. While our message included endorsements from political figures (i.e., “… Over 150 million vaccines have already been given to Americans, including presidents Trump and Biden …”) [57], we used a more ambiguous local referent (“…, and many people in your community.”) rather than coworkers, friends, family, or your healthcare provider. This might explain why our results deviate somewhat from recent findings [53, 54]. Yet, it also highlights the challenge of manipulating social norm cues through realistic and ethical endorsements if the vaccination behaviors of these trusted sources cannot be independently verified.

Constructing a good counterfactual is pivotal for causally robust tests of the effectiveness of nudges. To avoid experimenter demand effects and avoid confounding quantitative and qualitative elements of our treatment, all respondents watched a video of similar length, look, and feel. Videos also delivered the same basic sentiment: Get vaccinated against COVID-19. In an alternative universe, we could have made our control group respondents simply answer the questionnaire without watching a video. Yet, this approach would make it impossible to differentiate the effect of watching the video from the effectiveness of the behavioral science insights used to design its messages. By only varying the substantive appeal designed to nudge vaccination, but keeping everything else identical, we are able to isolate the effect of our messages and detect the kinds of appeals that reduce vaccine hesitancy and encourage willingness to get vaccinated.

It is important to interpret these results in light of our study’s limitations. First, our results are based on a non-representative sample of the US population. This is a common challenge in existing research. For instance, in studies of text reminders as tools to increase vaccine uptake [17], subjects were individuals who had already scheduled an appointment to get vaccinated. While our study might suffer some of the same limitations (e.g., people more interested in vaccines being more likely to self-select into a survey about vaccines), we were able to recruit a large, and fairly diverse sample of US adults as evidenced in S1 Appendix.

Second, we were unable to observe whether our messages increased actual uptake of COVID-19 vaccines. While we demonstrate that our video messages increased vaccination intentions, and that vaccination intentions correlated strongly with future uptake, research should seek to test our messages in field settings that allow for direct observation of actual vaccine uptake. This is particularly important in lieu of Dai et al.’s recent findings that a 2-minute video intervention produced a statistically significant effect on vaccination intentions, but failed to amplify the effect of a reminder text message on actual vaccine uptake [21]. Similarly, we cannot dismiss the notion that the controlled environment of our experimental study may have directed greater attention to the messages delivered as part of our videos than we would expect in natural settings. Dai and colleagues highlight this concern, noting that only 21% of individuals opted to watch the video in their field trial. However, subjects may not have felt a need to watch a video after already being primed with a text reminder, and brief video-based messages may therefore offer complementary interventions when text-based reminders are not feasible or a clearly defined target group does not exist. Nonetheless, conclusions on the application of our scripts as part of real-world public health campaigns [e.g., 58] rely on additional testing in field settings (e.g., as part of media campaigns). As such, any cost-benefit valuation of our scripts for vaccine uptake also necessarily depends on compliance (e.g., whether people actively watch the message if broadcasted on TV or as part of YouTube ads) and the extent to which intentions to vaccinate translate into actual vaccination behavior outside the lab settings.

Our study should not be viewed in isolation, but as part of others who have heeded the call to expand our toolkit of nudges and behavioral interventions to fight the coronavirus pandemic and future health crises [59]. As such, our messages can be seen as complementary to other important initiatives for increasing vaccinations like lotteries, gift cards, and other vaccination incentives [60–63]. However, unlike pecuniary incentives, persuasive messages of encouragement have the potential, not only to increase vaccinations, but to alter individuals’ attitudes about vaccines. This critical shift towards more positive attitudes can be expected to create down-stream effects, making individuals more likely to get vaccine boosters or future vaccines more generally, even in the absence of financial incentive programs. This is particularly important given concerns that populations may start to respond strategically to vaccination efforts as a function of past vaccination incentives or see incentives as coercive factors that may crowd out intrinsic motivations for vaccination [60, 64].

While more work is needed to address the concerns of the “moveable middle” [65, 66]–i.e., those whose vaccine perceptions lie someone between “accepts all vaccines” and “refuses all vaccines” (e.g., anti-vaxxers) on a vaccine acceptability spectrum–, our results offer encouraging and specific guidance to governments and health authorities on how to create effective messaging for COVID-19 vaccination campaigns. Our short video-based messages emphasizing (a) vaccine safety; (b) the efficacy of the vaccine to impact the pandemic’s social, economic, and public health trajectory; and (c) social norms to get vaccinated, (d) the ease, convenience, and ability to get the vaccine reduced vaccine hesitancy and boosted vaccination intentions for an estimated potential increase in future vaccination uptake of 5.7%. This effect was even larger among the most skeptical groups with an estimated boost upwards of 6.0% among politically conservatives and 9.0% among individuals expressing low trust in government institutions. While successful scripts should be tested at a large scale in natural field settings, they offer encouraging and specific lessons for designing COVID-19 vaccination campaigns to increase inoculation rates, as well as crucial lessons for addressing future health crises through nudge interventions.

## Supporting information

S1 Appendix Data collection, recruitment and data quality protocol. (PDF)

S2 Appendix Panel attrition analyses. (PDF)

S3 Appendix Verification of vaccination status. (PDF)

S4 Appendix Survey flowcharts. (PDF)

S5 Appendix Experimental video-based vignettes. (PDF)

S6 Appendix Estimation strategy. (PDF)

S1 Table Summary statistics for sample not fully vaccinated at T2. (PDF)

S2 Table Summary statistics for full sample. (PDF)

S3 Table Correlation matrix for sample of not fully vaccinated individuals. (PDF)

S4 Table Increase in vaccination intentions (T2) after watching treatment videos. Respondents failing attention check included.
(PDF)

S5 Table Increase in vaccination intentions (T2) after watching treatment videos. OLS regressions.
(PDF)

S6 Table T2 vaccination status predicted by T1 vaccination intention and controls. OLS regressions.
(PDF)

S7 Table Increase in self-efficacy (T2) after watching treatment videos. OLS regressions.
(PDF)

S8 Table Increase in response efficacy (T2) after watching treatment videos. OLS regressions.
(PDF)

S9 Table Response efficacy and self-efficacy as psychological mechanisms driving effect of video messages on vaccination intentions. (PDF)

S10 Table Heterogenous treatment effect on vaccination intentions by political ideology. (PDF)

S11 Table Heterogenous treatment effect on vaccination intentions by trust in government institutions. (PDF)

## References

[1] Frenck RW Jr, Klein NP, Kitchin N, Gurtman A, Absalon J, Lockhart S, et al. Safety, immunogenicity, and efficacy of the BNT162b2 Covid-19 vaccine in adolescents. New England Journal of Medicine. 2021;385. doi: 10.1056/NEJMoa2107456 34043894 PMC8174030

[2] Haas EJ, Angulo FJ, McLaughlin JM, Anis E, Singer SR, Khan F, et al. Impact and effectiveness of mRNA BNT162b2 vaccine against SARS-CoV-2 infections and COVID-19 cases, hospitalisations, and deaths following a nationwide vaccination campaign in Israel: An observational study using national surveillance data. The Lancet. 2021;397(10287). doi: 10.1016/S0140-6736(21)00947-8 33964222 PMC8099315

[3] Mendez R, Rattner N. Half of Americans 12 and Older are Fully Vaccinated as Daily Covid Case Counts Remain Below 15,000. CNBC. 2021 Jun 9 [cited 2022 Jan 31]. Available from: https://www.cnbc.com/2021/06/09/covid-19-cases-deaths-vaccinations-daily-update.html

[4] CDC. COVID Data Tracker; 2022 [cited 2022 Jan 31]. Available from: https://covid.cdc.gov/covid-data-tracker/#vaccinations

[5] Rattner N. Covid Vaccinations are Slowing in the U.S. as Supply Outstrips Demand. How States are Targeting Who’s Left. CNBC. 2021 Apr 30 [cited 2022 Jan 31] Available from: https://www.cnbc.com/2021/04/30/covid-vaccinations-in-us-are-slowing-as-supply-outstrips-demand.html

[6] Neergaard L, Fingerhut H. Poll: Most in US Who Remain Unvaccinated Need Convincing. AP News. 2021 May 11 [cited: 2022 Jan 31]. Available from: https://apnews.com/article/coronavirus-pandemic-health-0f0b89c8060da6dcce74057d2324dc44

[7] CDC. What You Need to Know About Variants. 2021 Dec 13 [cited 2022 Jan 31]. Available from: https://www.cdc.gov/coronavirus/2019-ncov/variants/about-variants.html

[8] Sheeran P. Intention—behavior relations: A conceptual and empirical review. European Review of Social Psychology. 2002;12(1). doi: 10.1080/14792772143000003

[9] Press Associated. After More than 500 Days, Denmark has Ended its COVID Restrictions. NPR. 2021 Sep 10 [cited 2022 Jan 31]. Available from: https://www.npr.org/2021/09/10/1036136246/covid-denmark-eu-restrictions

[10] Bonardi J-P, Bris A, Brülhart M, Danthine JP, Jondeau E, Rohner D, et al. (2020). The Case for Reopening Economies by Sectors. Harvard Business Review. 2020 May 19 [cited 2022 Jan 31]. Available from: https://hbr.org/2020/05/the-case-for-reopening-economies-by-sector

[11] Rothman AJ, Salovey P. Shaping perceptions to motivate healthy behavior: The role of message framing. Psychological Bulletin. 1997;121(1). doi: 10.1037/0033-2909.121.1.3 9000890

[12] Yuan S, Chu H. Vaccine for yourself, your community, or your country? Examining audiences’ response to distance framing of COVID-19 vaccine messages. Patient Education and Counseling. 2021 Aug 25. doi: 10.1016/j.pec.2021.08.019 34479746 PMC8384529

[13] James EK, Bokemper SE, Gerber AS, Omer SB, Huber GA. Persuasive messaging to increase COVID-19 vaccine uptake intentions. Vaccine. 2021;39(49). doi: 10.1016/j.vaccine.2021.10.039 34774363 PMC8531257

[14] Jordan JJ, Yoeli E, Rand DG. Don’t get it or don’t spread it: Comparing self-interested versus prosocial motivations for COVID-19 prevention behaviors. Scientific reports. 2021;11(1). doi: 10.1038/s41598-021-97617-5 34642341 PMC8511002

[15] Francis DB, Cates JR, Wagner KPG, Zola T, Fitter JE, Coyne-Beasley T. Communication technologies to improve HPV vaccination initiation and completion: A systematic review. Patient Education and Counseling. 2017;100(7). doi: 10.1016/j.pec.2017.02.004 28209248

[16] Zimet G, Dixon BE, Xiao S, Tu W, Kulkarni A, Dugan T, et al. Simple and elaborated clinician reminder prompts for human papillomavirus vaccination: A randomized clinical trial. Academic Pediatrics. 2018;18(2). doi: 10.1016/j.acap.2017.11.002 29502640

[17] Milkman KL, Patel MS, Gandhi L, Graci HN, Gromet DM, Ho H. et al. A megastudy of text-based nudges encouraging patients to get vaccinated at an upcoming doctor’s appointment. Proceedings of the National Academy of Sciences. 2021;118(20). doi: 10.1073/pnas.2101165118 33926993 PMC8157982

[18] Regan AK, Bloomfield L, Peters I, Effler PV. Randomized controlled trial of text message reminders for increasing influenza vaccination. The Annals of Family Medicine. 2017;15(6). doi: 10.1370/afm.2120 29133488 PMC5683861

[19] Milkman KL, Beshears J, Choi JJ, Laibson D, Madrian BC. Using implementation intentions prompts to enhance influenza vaccination rates. Proceedings of the National Academy of Sciences. 2011;108(26). doi: 10.1073/pnas.1103170108 21670283 PMC3127912

[20] Yokum D, Lauffenburger JC, Ghazinouri R, Choudhry NK. Letters designed with behavioural science increase influenza vaccination in Medicare beneficiaries. Nature Human Behaviour. 2018;2(10). doi: 10.1038/s41562-018-0432-2 31406294

[21] Dai H, Saccardo S, Han MA, Roh L, Raja N, Vangala S, et al. Behavioural nudges increase COVID-19 vaccinations. Nature. 2021;597(7876). doi: 10.1038/s41586-021-03843-2 34340242 PMC8443442

[22] Keppeler F, Sievert M, Jilke S. How local government vaccination campaigns can increase willingness to get vaccinated against Covid-19: A field experiment on psychological ownership. SSRN:3905470 [Preprint]. 2021 [cited 2022 Jan 31]. Available from: 10.2139/ssrn.3905470

[23] Catalano HP, Knowlden AP, Birch DA, Leeper JD, Paschal AM, Usdan SL. Using the theory of planned behavior to predict HPV vaccination intentions of college men. Journal of American College Health. 2017;65(3). doi: 10.1080/07448481.2016.1269771 27960609

[24] Gerend MA, Shepherd JE. Predicting human papillomavirus vaccine uptake in young adult women: Comparing the health belief model and theory of planned behavior. Annals of Behavioral Medicine. 2012;44(2). doi: 10.1007/s12160-012-9366-5 22547155 PMC3439593

[25] Brewer NT, Chapman GB, Rothman AJ, Leask J, Kempe A. Increasing vaccination: Putting psychological science into action. Psychological Science in the Public Interest. 2017;18(3). doi: 10.1177/1529100618760521 29611455

[26] Betsch C, Schmid P, Heinemeier D, Korn L, Holtmann C, Böhm R. Beyond confidence: Development of a measure assessing the 5C psychological antecedents of vaccination. PLoS ONE. 2018;13(12). doi: 10.1371/journal.pone.0208601 30532274 PMC6285469

[27] Kennedy R, Clifford S, Burleigh T, Waggoner PD, Jewell R, Winter NJ. The shape of and solutions to the MTurk quality crisis. Political Science Research and Methods. 2020;8(4). doi: 10.1017/psrm.2020.6

[28] Litman L, Robinson J, Abberbock T. TurkPrime. com: A versatile crowdsourcing data acquisition platform for the behavioral sciences. Behavior Research Methods. 2017;49(2). doi: 10.3758/s13428-016-0727-z 27071389 PMC5405057

[29] Ajzen I. The theory of planned behavior. Organizational Behavior and Human Decision Processes. 1991;50(2). doi: 10.1016/0749-5978(91)90020-T

[30] Xiao X, Wong RM. Vaccine hesitancy and perceived behavioral control: A meta-analysis. Vaccine. 2020;38(33). doi: 10.1016/j.vaccine.2020.04.076 32409135

[31] Shmueli L. Predicting intention to receive COVID-19 vaccine among the general population using the health belief model and the theory of planned behavior model. BMC Public Health. 2021;21(1). doi: 10.1186/s12889-021-10816-7 33902501 PMC8075011

[32] CDC. Understanding the Federal Retail Pharmacy Program for COVID-19 Vaccination. 2021 Dec 27 [cited 2022 Jan 31]. Available from: https://www.cdc.gov/vaccines/covid-19/retail-pharmacy-program/index.html.

[33] Tur B, Harstad J, Antonakis J. Effect of charismatic signaling in social media settings: Evidence from TED and Twitter. The Leadership Quarterly. 2021 Feb 27:101476. doi: 10.1016/j.leaqua.2020.101476

[34] Jensen UT, Rohner D, Bornet O, Carron D, Garner P, Loupi D, et al. Combating COVID-19 with charisma: Evidence on governor speeches and physical distancing in the United States. PsyArXiv. [Preprint]. 2021 [cited 2022 Jan 31]. Available from: 10.31234/osf.io/ypqmk

[35] Zizzo DJ. Experimenter demand effects in economic experiments. Experimental Economics. 2010;13(1). doi: 10.1007/s10683-009-9230-z

[36] Funk C, Tyson A. Growing Share of Americans Say They Plan to Get a COVID-19 Vaccine–or Already Have. Pew Research Center. 2021 Mar 5 [cited 2022 Jan 31]. Available from: https://www.pewresearch.org/science/2021/03/05/growing-share-of-americans-say-they-plan-to-get-a-covid-19-vaccine-or-already-have/.

[37] Mundell E, Foster R. U.S. Officials Say 50% of American Adults are Now Fully Vaccinated. US News. 2021 May 26 [cited 2022 Jan 31]. Available from: https://www.usnews.com/news/health-news/articles/2021-05-26/us-officials-say-50-of-american-adults-are-now-fully-vaccinated.

[38] Wickens TD, Keppel G. Design and analysis: A researcher’s handbook. Upper Saddle River, NJ: Pearson Prentice-Hall; 2004.

[39] MacDonald NE. Vaccine hesitancy: Definition, scope and determinants. Vaccine. 2015;33(34). doi: 10.1016/j.vaccine.2015.04.036 25896383

[40] Sajons GB. Estimating the causal effect of measured endogenous variables: A tutorial on experimentally randomized instrumental variables. The Leadership Quarterly. 2020;31 (5). doi: 10.1016/S1048-9843(20)30091-6 32982126 PMC7508016

[41] Jones J. COVID-19 Vaccine-Reluctant in U.S. Likely to Stay that Way. Gallup. 2021 Jun 7 [cited 2022 Jan 31]. Available from: https://news.gallup.com/poll/350720/covid-vaccine-reluctant-likely-stay.aspx.

[42] Gollwitzer A, Martel C, Brady WJ, Pärnamets P, Freedman IG, Knowles ED, et al. Partisan differences in physical distancing are linked to health outcomes during the COVID-19 pandemic. Nature Human Behavior. 2020;4. doi: 10.1038/s41562-020-00977-7 33139897

[43] Kerr J, Panagopoulos C, van der Linden S. Political polarization on COVID-19 pandemic response in the United States. Personality and Individual Differences. 2021;179. doi: 10.1016/j.paid.2021.110892 34866723 PMC8631569

[44] Everett JA, Colombatto C, Awad E, Boggio P, Bos B, Brady WJ, et al. Moral dilemmas and trust in leaders during a global health crisis. Nature Human Behaviour. 2021;5(8). doi: 10.1038/s41562-021-01156-y 34211151

[45] Lindholt MF, Jørgensen F, Bor A, Petersen MB. Public acceptance of COVID-19 vaccines: cross-national evidence on levels and individual-level predictors using observational data. BMJ Open. 2021;11(6). doi: 10.1136/bmjopen-2020-048172 34130963 PMC8210695

[46] Bach AT, Goad JA. The role of community pharmacy-based vaccination in the USA: Current practice and future directions. Integrated Pharmacy Research & Practice. 2015;4. doi: 10.2147/IPRP.S63822 29354521 PMC5741029

[47] Shah PD, Marciniak MW, Golden SD, Trogdon JG, Golin CE, Brewer NT. Pharmacies versus doctors’ offices for adolescent vaccination. Vaccine. 2018;36(24). doi: 10.1016/j.vaccine.2018.04.088 29748030

[48] Sharfstein JM, Callaghan T, Carpiano RM, Sgaier SK, Brewer NT, Galvani AP, et al. Uncoupling vaccination from politics: A call to action. The Lancet. 2021;398(10307). doi: 10.1016/S0140-6736(21)02099-7 34537104 PMC8445735

[49] Armitage CJ, Conner M. Efficacy of the theory of planned behaviour: A meta‐analytic review. British Journal of Social Psychology. 2001;40(4). doi: 10.1348/014466601164939 11795063

[50] Pfattheicher S, Petersen MB, Böhm R. Information about herd immunity through vaccination and empathy promote COVID-19 vaccination intentions. Health Psychology. Forthcoming. doi: 10.1037/hea0001096 34570535

[51] Motta M, Sylvester S, Callaghan T, Lunz-Trujillo K. Encouraging COVID-19 vaccine uptake through effective health communication. Frontiers in Political Science. 2021;3. doi: 10.3389/fpos.2021.630133

[52] Zampetakis LA, Melas C. The health belief model predicts vaccination intentions against COVID‐19: A survey experiment approach. Applied Psychology: Health and Well‐Being. 2021;13(2). doi: 10.1111/aphw.12262 33634930 PMC8014148

[53] Moehring A, Avinash C, Garimella K, Rahimian MA, Aral S, Eckles D. Surfacing norms to increase vaccine acceptance. SSRN:3705470 [Preprint]. 2021 [cited 2022 Jan 31]. Available from: 10.2139/ssrn.3782082

[54] Sinclair S, Agerström J. Do social norms influence young people’s willingness to take the COVID-19 vaccine? Health Communication. 2021 Forthcoming. doi: 10.1080/10410236.2021.1937832 34114897

[55] Li M, Taylor EG, Atkins KE, Chapman GB, Galvani AP. Stimulating influenza vaccination via prosocial motives. PloS ONE. 2016;11(7). doi: 10.1371/journal.pone.0159780 27459237 PMC4961402

[56] Four Messages that can Increase Uptake of the COVID-19 Vaccines. 2021 Mar 15 [cited Jan 31 2022]. In: The Behavioural Insights Team Blog [Internet]. Available from: https://www.bi.team/blogs/four-messages-that-can-increase-uptake-of-the-covid-19-vaccines/.

[57] Pink SL, Chu J, Druckman J, Rand DG, Willer R. Elite party cues increase vaccination intentions among republicans. Proceedings of the National Academy of Sciences. 2021;118(32). doi: 10.1073/pnas.2106559118 34312254 PMC8364165

[58] Breza E, Stanford FC, Alsan M, Alsan B, Banerjee A, Chandrasekhar AG, et al. Effects of a large-scale social media advertising campaign on holiday travel and COVID-19 infections: A cluster randomized controlled trial. Nature Medicine. 2021;27(9). doi: 10.1038/s41591-021-01487-3 34413518 PMC8440209

[59] Betsch C, Wieler LH, Habersaat K. Monitoring behavioural insights related to COVID-19. The Lancet. 2020;395(10232). doi: 10.1016/S0140-6736(20)30729-7 32247323 PMC7163179

[60] Volpp KG, Cannuscio CC. Incentives for immunity—Strategies for increasing Covid-19 vaccine uptake. New England Journal of Medicine. 2021;385:e1. doi: 10.1056/NEJMp2107719 34038633

[61] Klüver H, Hartmann F, Humphreys M, Geissler F, Giesecke J. Incentives can spur COVID-19 vaccination uptake. Proceedings of the National Academy of Sciences. 2021;118(36). doi: 10.1073/pnas.2109543118 34413212 PMC8433545

[62] Campos-Mercade P, Meier AN, Schneider FH, Meier S, Pope D, Wengström E. Monetary incentives increase COVID-19 vaccinations. Science. 2021;374(6569). doi: 10.1126/science.abm0475 34618594 PMC10765478

[63] Wilf-Miron R, Myers V, Saban M. Incentivizing vaccination uptake: The “green pass” proposal in Israel. JAMA. 2021;325(15). doi: 10.1001/jama.2021.4300 33720271

[64] Largent EA, Miller FG. Problems with paying people to be vaccinated against COVID-19. JAMA. 2021;325(6). doi: 10.1001/jama.2020.27121 33404585

[65] Attwell K, Lake J, Sneddon J, Gerrans P, Blyth C, Lee J. Converting the maybes: Crucial for a successful COVID-19 vaccination strategy. PLoS ONE. 2021;16(1). doi: 10.1371/journal.pone.0245907 33471821 PMC7817004

[66] Alsabbagh MW, Church D, Wenger L, Papastergiou J, Raman-Wilms L, Schneider E, et al. Pharmacy patron perspectives of community pharmacist administered influenza vaccinations. Research in Social and Administrative Pharmacy. 2019;15(2). doi: 10.1016/j.sapharm.2018.04.015 29724679

