# Supplementary material for: Video-based messages to reduce COVID-19 vaccine hesitancy and nudge vaccination intentions
Source: PLoS One. 2022 Apr 6;17(4):e0265736. doi: 10.1371/journal.pone.0265736 (PMC8985948; doi:10.1371/journal.pone.0265736)
Supplement: S1 Appendix — (PDF) [file pone.0265736.s001.pdf]

S1 Appendix. Data Collection, Recruitment and Data Quality Protocol

Amazon's Mechanical Turk (Mturk) is an online labor marketplace widely used by social and behavioral scientists to recruit and pay respondents for survey and experimental research as well as other crowdsourced tasks [1]. Mturk provides immediate access to a large and diverse work-for-hire respondent pool with a majority of its workers ("turkers") based in the United States.

With its proliferation as a platform for data collection, several criticism and concerns have been voiced. Among the most notable concerns are poor data quality and limited external validity. Below we outline each of the major concerns identified by past research, and how we implemented robust protocols to mitigate each of these challenges and create a high-quality sample for our study.

<u>Concern 1:</u> Poor data quality due to non-human responses like bots engineered to complete simple survey tasks.

<u>Remedy 1:</u> We included a reCAPTCHA as the first part of our survey. This technology imposes a visual recognition over multiple iterations based on keywords and is widely used as a mechanism for detecting and screening out non-human online behavior.

<u>Concern 2:</u> Poor data quality due to international respondents using VPN or VPS technology to mask their geolocation and game study inclusion criteria.

<u>Remedy 2:</u> We implemented the protocol by [2] Kennedy et al. (2020) to screen out international respondents using a VPN/VPS. We used the hidden timer function in Qualtrics to require all respondents spend at least 20 seconds on our consent page. During these 20 seconds, a JavaScript stripped the respondent's IP address and verified it against known IP addresses using a third-party service (IPHub). If an IP could not be identified, respondents were prompted to manually enter their Amazon Mturk unique worker identifier for post-hoc verification by the research team.

<u>Concern 3:</u> Poor data quality due to lack of attention and care in responding.

<u>Remedy 3:</u> We took several steps to mitigate this concern. First, we included an activity-based attention check asking respondents' how often "they eat cement?". To make it difficult to detect, we disguised it as part of a larger battery of questions. 78 respondents failed to answer "never" and were screened out (see SI2 for more details).

Second, we included two attention checks as the last questions in our survey. Respondents were asked to recall the gender of the actor in the video ("**man**" or

“woman”), and recall the public health behavior encouraged in the video (“**getting a COVID-19 vaccine**”, “making sure you wear a mask over both nose and mouth”, or “avoiding other people”).

Finally, we made sure to offer respondents fair compensation for their time and effort. Respondents were provided a base compensation of $0.50 and average bonus payout of $0.56 for an average total compensation of $1.06. We expected responses to take ~5 minutes, yielding an hour rate of $12.70. 261 (or ~30% of sample) completed the survey in 5 minutes or less. The median response time was approximately 8 minutes and 30 seconds, still yielding an hourly rate ($7.50) that exceeds the US federal minimum wage of $7.25.

Concern 4: Limited external validity due to poor demographic representation.

Remedy 4: While certain populations are overrepresented on Mturk as discussed below, scholars have shown that results from replication experiments performed on Mturk are largely similar to those obtained from national samples (e.g., [3] Coppock 2019). These results support an emerging consensus that convenience samples drawn from Mturk allow researchers to produce externally valid experimental findings (e.g., [1] Buhrmester et al., 2011; [4] Mullinix et al., 2015; [5] Paolacci et al., 2010).

While this supports that our experimental findings are unbiased and externally valid, concerns over generalizability might still loom large if our sample is markedly different from the general population. Below, we compare our full sample (i.e., everyone who completed T2 survey) and the subsample of individuals who were not fully vaccinated at T2 against US census information on different demographic characteristics.

Table A Sample vs. population summary statistics on demographic characteristics

Variable	Sample (All)	Sample (Not fully vaccinated)	US Population
Gender (% Men)	51.1	49.4	49.2[a]
Age (Mean)	42.3	40.7	38.2[a,b]
Race/Ethnicity (% White)	76.3	73.4	60.1[a]
Education (% with College Degree)	54.4	50.1	32.1[a]
Urban/Rural Living (% in Urban Areas)	76.5	74.5	81.0[a]
Political Ideology (%)			
Conservative	35.7	46.1	36.0[c]
Moderate	21.6	21.9	35.0[c]
Liberal	42.7	32.0	25.0[c]
Vaccination Intention (% Willing to get Vaccinated)			
T1 (January/February 2021)	68.6	49.1	71.0[d]

T2 (May 2021)	73.0	50.3	76.0[d]
Vaccination Uptake - T2 (% vaccinated)	57.2	-	61.6[e]

Notes: a [6], b [7]. Note that census is for the entire US population, while our sample only include adults 18 years of age and older, c [8], d [9], e [10].

Previous research has reported an overrepresentation of younger, higher educated, white and liberal populations on Mturk. This is the case for our sample as well. However, it is notable that our samples matches the general population on several key characteristics such as gender, rural/urban living, politically conservative, and on vaccination intentions and uptake. Existing research echoes the validity of Mturk as a platform for recruiting convenience samples for social science research (e.g., [11] Berinsky, Huber and Lenz 2012). Levay, Freese and Druckman [12] (2016), for instance, show that Mturk respondents do not differ fundamentally from population-based respondents in unmeasurable ways after controlling for sample features. Nonetheless, our sample is overrepresented by liberal, higher educated and white individuals, all demographic groups expressing greater vaccination intentions, and interpretations of our results should therefore take these issues into account.

We recruited respondents through CloudResearch [13]. CloudResearch allows researchers to source respondents' Amazon worker id by tagging it onto the unique URL string for the survey links. We leveraged this feature to match responses over time, and limit inclusion for the second survey based on respondents' unique identifier.

References:

1 Buhrmester, M., Kwang, T., & Gosling, S. D. (2011). Amazon's Mechanical Turk: A new source of inexpensive, yet high-quality, data? *Perspectives on Psychological Science*, 6, 3-5.

2 Kennedy, R., Clifford, S., Burleigh, T., Waggoner, P., Jewell, R., & Winter, N. (2020). The shape of and solutions to the MTurk quality crisis. *Political Science Research and Methods*, 8(4), 614-629.

3 Coppock, A. (2019). Generalizing from survey experiments conducted on Mechanical Turk: A replication approach. *Political Science Research and Methods*, 7(3), 613-628.

4 Mullinix, K. J., Leeper, T. J., Druckman, J. N., & Freese, J. (2015). The generalizability of survey experiments. *Journal of Experimental Political Science*, 2(2), 109-138.

5 Paolacci, G., & Chandler, J. (2014). Inside the Turk: Understanding Mechanical Turk as a participant pool. *Current Directions in Psychological Science*, 23(3), 184-188.

6 U.S. Census. https://www.census.gov/quickfacts/fact/table/US/PST045219.

7 U.S. Census. https://mtgis-portal.geo.census.gov/arcgis/apps/MapSeries/index.html?appid=49cd4bc9c8eb444ab51218c1d5001ef6.

8 Gallup Research. https://news.gallup.com/poll/328367/americans-political-ideology-held-steady-2020.aspx.

9 Gallup Research. https://news.gallup.com/poll/350720/covid-vaccine-reluctant-likely-stay.aspx.

10 Centers for Disease Control and Prevention. (2021). COVID Data Tracker. Accessed June 16, 2021: https://covid.cdc.gov/covid-data-tracker/#vaccinations.

11 Berinsky, A. J., Huber, G. A., & Lenz, G. S. (2012). Evaluating online labor markets for experimental research: Amazon.com's Mechanical Turk. *Political Analysis*, 20(3), 351-368.

12 Levay, K. E., Freese, J., & Druckman, J. N. (2016). The demographic and political composition of Mechanical Turk samples. *Sage Open*, 6(1), 2158244016636433.

13 Litman, L., Robinson, J., & Abberbock, T. (2017). TurkPrime.com: A versatile crowdsourcing data acquisition platform for the behavioral sciences. *Behavior Research Methods*, 49(2), 433-442.
