# Supplementary material for: Video-based messages to reduce COVID-19 vaccine hesitancy and nudge vaccination intentions
Source: PLoS One. 2022 Apr 6;17(4):e0265736. doi: 10.1371/journal.pone.0265736 (PMC8985948; doi:10.1371/journal.pone.0265736)
Supplement: S2 Appendix — (PDF) [file pone.0265736.s002.pdf]

S2 Appendix. Panel Attrition Analyses

The following analyses explored whether the subset (*n* = 890) of respondents who completed both surveys (T1 and T2) differ in systematic ways from respondents who only completed the first (T1) survey. Figure S2 shows that there is a systematic difference between those completing both surveys and those who did not: $F(8, 1447) = 7.51$, $p < 0.001$ and $F(14, 1441) = 4.94$, $p < 0.001$, for models 1 and 2 respectively.

However, a closer inspection clearly reveals that these differences are driven almost exclusively by large difference between these subsets of respondents along age (beta = 0.008, $t = 7.10$, cf. Model 2). Older respondents were thus more likely to complete the second survey compared to younger respondents. We see a similar trend for respondents with a college degree compared to only a high school degree. However, we see no differences along gender, race/ethnicity, political ideology, initial vaccination intentions or a host of vaccine attitudes and beliefs. With a 60.5% retention rate, and observable differences along only one or two characteristics, attrition seems to be mostly random, or the product of a single, idiosyncratic respondent characteristic.

Table A Predicting follow-up survey (T2) completion based on baseline (T1) characteristics

	Model 1	Model 2
Man *(Ref. = Woman)*	0.043	0.043
	(1.69)	(1.70)
Age	0.008**	0.008**
	(7.15)	(7.10)
Education *(Ref. = High School)*		
College	0.075**	0.068*
	(2.68)	(2.41)
Professional Degree	0.08	0.074
	(1.93)	(1.76)
Doctorate	0.016	-0.004
	(0.11)	(-0.03)
White *(Ref. = Non-White)*	-0.025	-0.029
	(-0.81)	(-0.94)
Political Ideology *(Ref. = Liberal)*		
Moderate	0.009	0.015
	(0.26)	(0.42)
Conservative	-0.003	0.001
	(-0.10)	(0.05)
Vaccination Intention		-0.010

		(-1.73)
Self-Efficacy		0.006
		(1.26)
Response Efficacy		0.009
		(1.63)
Safety Concern		-0.004
		(-0.59)
Desire to Protect		0.005
		(0.95)
Social Norm Belief		-0.014
		(-1.70)
Constant	0.243**	0.222*
	(4.36)	(2.37)
Observations	1,456	1,456
R-squared	0.04	0.05
F-test	$F(8, 1447) = 7.51, p < 0.001$	$F(14, 1441) = 4.94, p < 0.001$

Notes: ** p<0.01, * p<0.05. OLS regressions with indicator for T2 sample membership as dependent variable. T-statistics in parentheses.
