# Supplementary material for: Video-based messages to reduce COVID-19 vaccine hesitancy and nudge vaccination intentions
Source: PLoS One. 2022 Apr 6;17(4):e0265736. doi: 10.1371/journal.pone.0265736 (PMC8985948; doi:10.1371/journal.pone.0265736)
Supplement: S3 Appendix — (PDF) [file pone.0265736.s003.pdf]

S3 Appendix. Verification of Vaccination Status

To verify vaccination status, we incentivized respondents who reported being vaccinated to share a redacted version of their CDC vaccination card with us. Specifically, respondents were shown the following prompt:

"Earlier you indicated that you have received a COVID-19 vaccine. Everyone who has been vaccinated against COVID-19 in the U.S. has received a vaccination card from your local health authorities and the CDC that tells you what COVID-19 vaccine you received, the date you received it, and where you received it. The card looks like the one shown below. If you share a photo of your card with us, we will compensate you an added bonus of **$1.00 USD**. We will only provide the added bonus if you follow all instructions below."

Respondents were then instructed to: (1) only take a photo of the frontpage of their card, (2) place a finger or object (like a pen) over name and birth to redact any person-identifying information, (3) to write down their unique Amazon Mturk worker ID on a small note or piece of paper and place it next to the CDC card. This is crucial as it allows match vaccination status to be linked to survey responses and guard against manipulating or fabricating vaccination status. We provided the following example to make instructions clear and easily understandable.

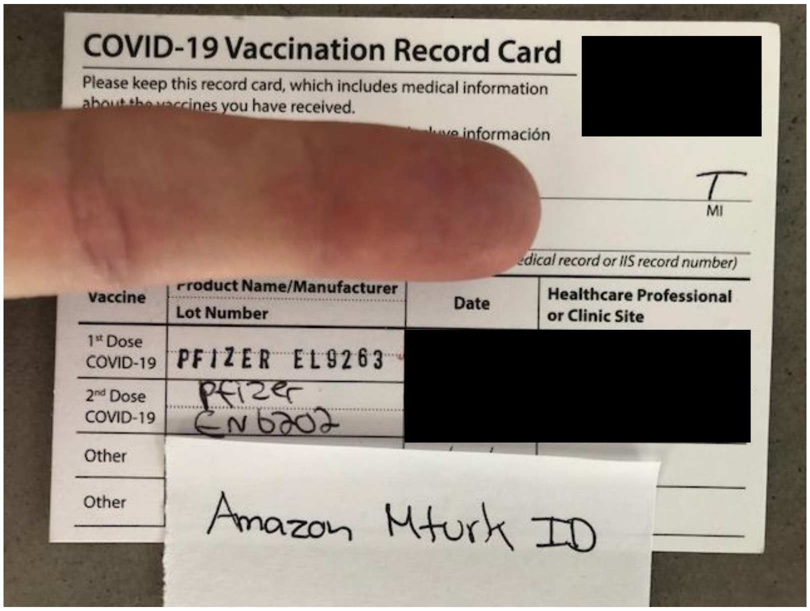

Fig. A. Redacted CDC vaccination card provided as example to survey respondents. Picture is similar but not identical to the actual picture provided to study respondents. In the study, date of

inoculation, site, and logos were not redacted.

Finally, we provided a disclaimer to respondents stating that photos were only to be used for the purpose of our research study, would not be shared with anyone outside the research team, and would be stored on password protected server.
