# Supplementary material for: Video-based messages to reduce COVID-19 vaccine hesitancy and nudge vaccination intentions
Source: PLoS One. 2022 Apr 6;17(4):e0265736. doi: 10.1371/journal.pone.0265736 (PMC8985948; doi:10.1371/journal.pone.0265736)
Supplement: S4 Appendix — (PDF) [file pone.0265736.s004.pdf]

S4 Appendix. Survey Flowcharts

Fig. A. Baseline (T1) survey flowchart

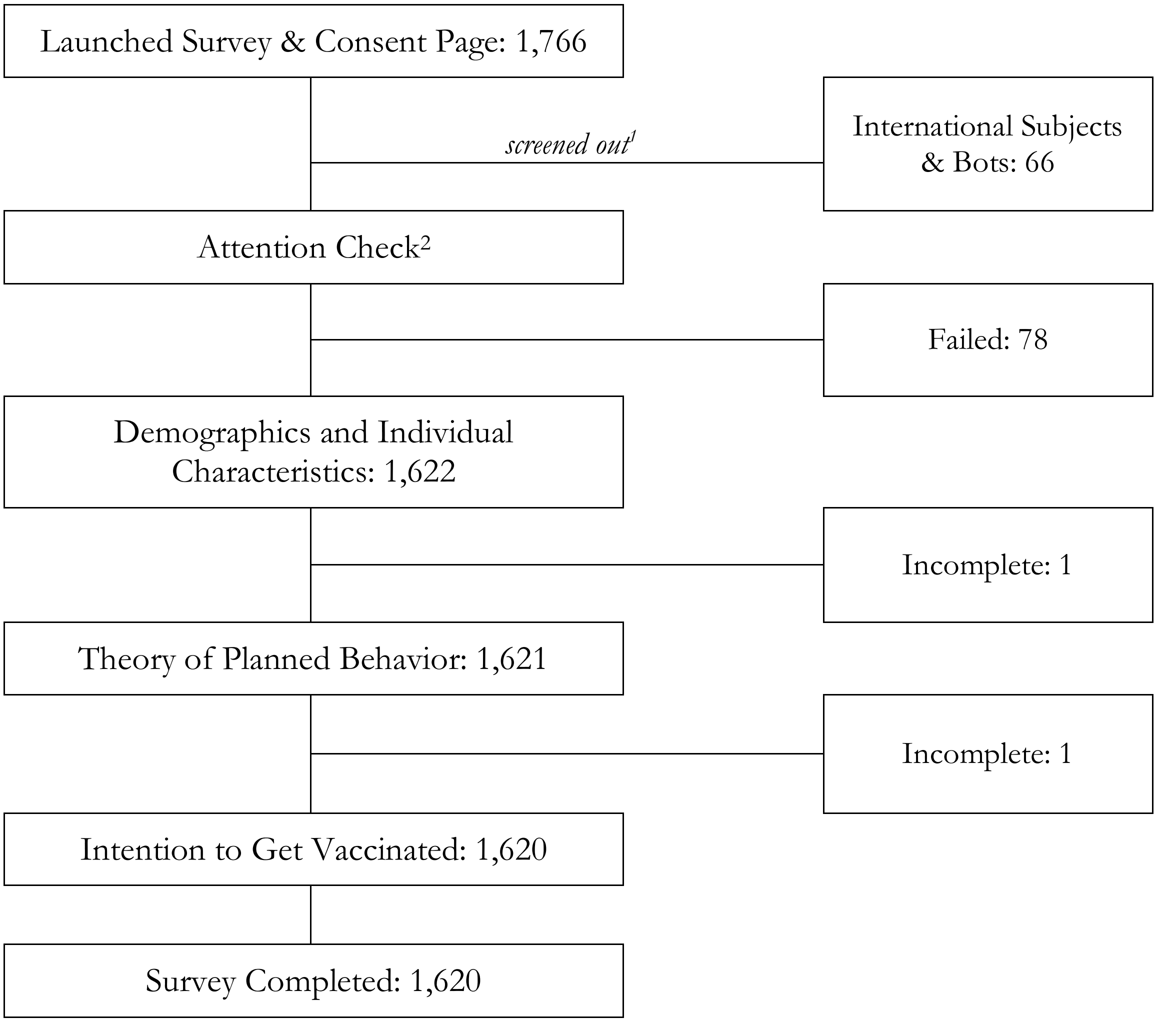

[1] We followed Kennedy et al.'s (2020) protocol for screening out international respondents using a VPN/VPS to mask their geolocation. All respondents were required to spend at least 20 seconds reading the consent page as an intro to the survey. During these 20 seconds, a JavaScript stripped the respondent's IP address and ran it against known IP addresses using a third-party service (IPHub). Second, the study used a recaptcha verification to screen out bots and automated non-human respondents. Taken together this protocol boosts our confidence in the quality of the data and helps enforce our exclusion criteria.

2 Disguised as part of the inventory of Theory of Planned Behavior questions, respondents were asked "how often do you eat cement?" with the following response options: "never", "rarely", "sometimes", "often" or "always". All non-never responses (n = 78) were categorized as an indicator of inattentive respondents.

Fig. B. Follow-up (T2) survey flowchart

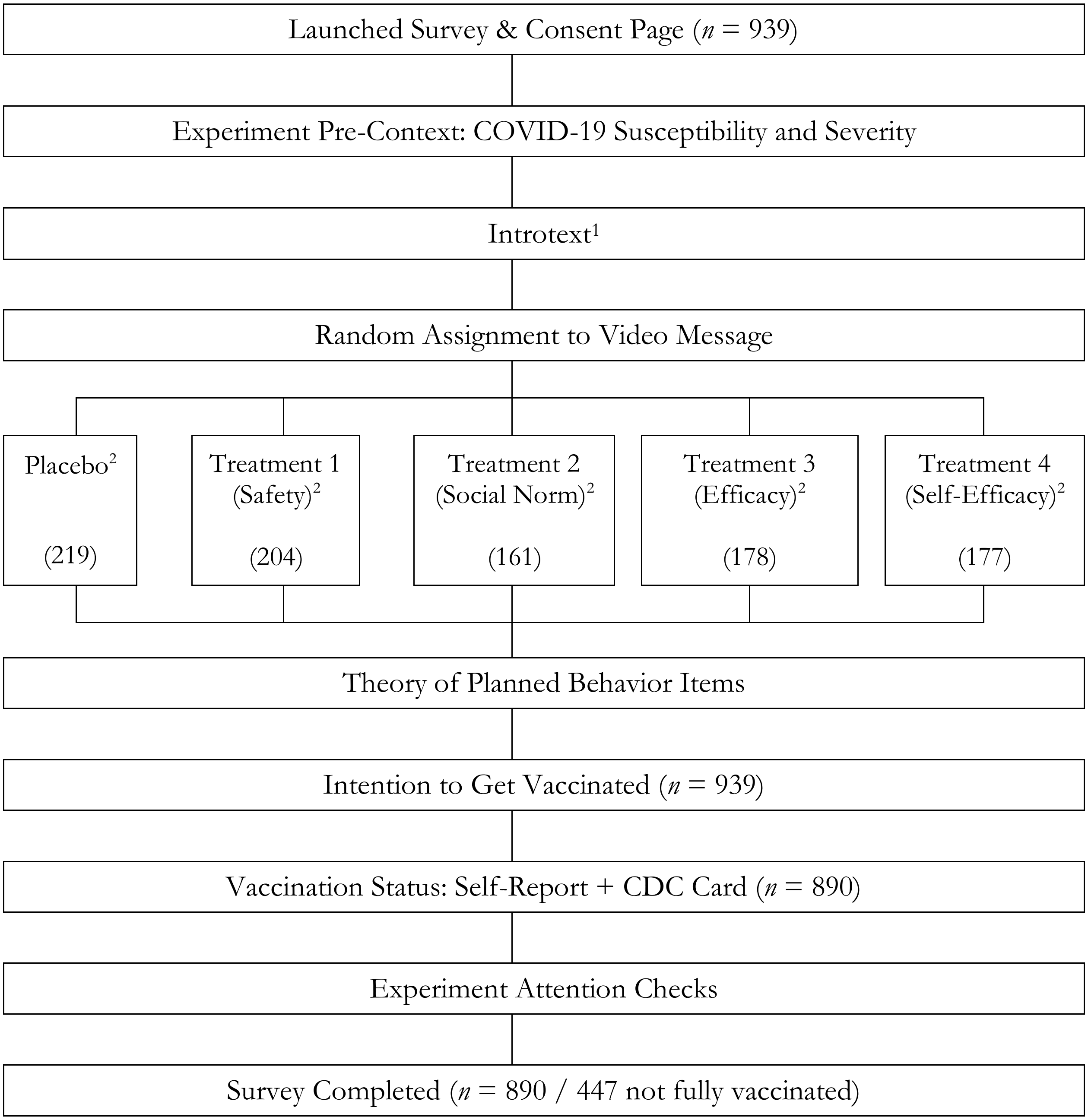

1 The following introductory text was presented to all subjects: "You are now going to be presented with some information about the current coronavirus pandemic and vaccines developed to protect against the virus. Please pay careful attention to the information presented to you and provide your immediate and honest opinions.

We will first show you a short video. Pay close attention to the information provided in the video, because we will ask you questions on it later that can affect your payout. After the video is done, you will automatically advance to the next question."

2 Video messages varied in length between 29 and 32 seconds. Detailed description and full transcripts of all videos are presented in S5 Appendix.

For all videos, the Qualtrics page submit button was delayed with the video length time plus two seconds. Subjects automatically advanced after the video length time plus one second ensuring no respondent could skip ahead before watching the entire video.

Finally, we hid all video control functions and programmed videos to auto play as soon as the Qualtrics page loaded. This helped ensure that all subjects were exposed to the full duration of the videos.
