# Supplementary material for: Video-based messages to reduce COVID-19 vaccine hesitancy and nudge vaccination intentions
Source: PLoS One. 2022 Apr 6;17(4):e0265736. doi: 10.1371/journal.pone.0265736 (PMC8985948; doi:10.1371/journal.pone.0265736)
Supplement: S5 Appendix — (PDF) [file pone.0265736.s005.pdf]

S5 Appendix. Experimental Video-based Vignettes

Five video messages make up the experimental conditions for manipulating drivers for getting vaccinated against COVID-19 (safety concern, social norm, response efficacy, self-efficacy, and placebo).

All videos feature the same male actor portraying a pharmacist. An appropriate disclaimer noting "actor portrayal" is issued on all videos. All videos contain identical intro and exit sections, with only middle sections varying between conditions. Each video is presented below along with brief meta-information.

Movie A. Condition 1 – motivational encouragement (placebo)

Hi. My name is Scott Schmidt. Did you know? COVID-19 vaccines work like a commander, telling our immune system how to recognize and fight the virus. Getting a vaccine is important, and it is recommended even if you have already recovered from COVID-19 because you could get sick again. So, what are you going to do? Look for information from your local health authorities and pharmacies on how to schedule your vaccine.

Link: https://youtu.be/XPQWKCYws4M

Meta-information: Total words: 72. Number of charismatic communication tactics: 3.75.[1] Source: Centers for Disease Control and Prevention's Myths and Facts about COVID-19 vaccines: https://www.cdc.gov/coronavirus/2019-ncov/vaccines/facts.html.

Movie B. Condition 2 – vaccine safety (treatment 1)

Hi. My name is Scott Schmidt. Did you know? Millions have received COVID-19 vaccines, and thousands have been tested and monitored for side effects to prove its safety. In comparison, your biggest risk is dying from everyday activities like driving, not from developing severe allergic reactions to COVID-19 vaccines. So, what are you going to do? Look for information from your local health authorities and pharmacies on how to schedule your vaccine.

Link: https://youtu.be/tTnfTgNHhe0

Meta-information: Total words: 72. Number of charismatic communication tactics: 3.99. [1] Sources: https://www.cdc.gov/coronavirus/2019-ncov/vaccines/safety/adverse-events.html and https://www.usatoday.com/story/money/cars/2021/03/05/pandemic-travel-traffic-deaths-up-8-2020-despite-driving-less/4590942001/

Movie C. Condition 3 – social norm (treatment 2)

Hi. My name is Scott Schmidt. Did you know? 7 out of 10 Americans want to get a COVID-19 vaccine to protect their loved ones and their community. Over 150 million vaccines have already been given to Americans, including presidents Trump and Biden, and many people in your community. So, what are you going to do? Look for information from your local health authorities and pharmacies on how to schedule your vaccine.

Link: https://youtu.be/mMugpb63qrM

Meta-information: Total words: 72. Number of charismatic communication tactics: 3.64. [1] Sources: https://www.pewresearch.org/science/2021/03/05/growing-share-of-americans-say-they-plan-to-get-a-covid-19-vaccine-or-already-have/ and https://www.npr.org/sections/health-shots/2021/01/28/960901166/how-is-the-covid-19-vaccination-campaign-going-in-your-state.

Movie D. Condition 4 – response efficacy (treatment 3)

Hi. My name is Scott Schmidt. Did you know? Vaccines are so effective that the risk of getting infected with COVID-19 is reduced 90% after two doses. Vaccines are the number one healthcare resource for stopping the pandemic, fully re-opening the economy, and getting us back to our pre-COVID "normal". So, what are you going to do? Look for information from your local health authorities and pharmacies on how to schedule your vaccine.

Link: https://youtu.be/jKROSDIzK6s

Meta-information: Total words: 73. Number of charismatic communication tactics: 3.97. [1] Sources: https://www.cdc.gov/vaccines/acip/recs/grade/covid-19-pfizer-biontech-vaccine.html, https://www.cdc.gov/vaccines/acip/recs/grade/covid-19-moderna-vaccine.html,

https://www.cdc.gov/mmwr/volumes/70/wr/mm7013e3.htm?s_cid=mm7013e3_w#contribAff, and https://www.cdc.gov/media/releases/2021/p0329-COVID-19-Vaccines.html

Movie E. Condition 5 – self-efficacy (treatment 4)

Hi. My name is Scott Schmidt. Did you know? COVID-19 vaccines are free for all Americans and paid for by our government. Pharmacies are delivering COVID-19 vaccines, making it convenient and flexible for you to get your vaccine without having to drive far or wait in a long line. So, what are you going to do? Look for information from your local health authorities and pharmacies on how to schedule your vaccine.

Link: https://youtu.be/iBr_SElK6V4

Meta-information: Total words: 72. Number of charismatic communication tactics: 3.26. [1] Source: https://www.cnet.com/personal-finance/the-covid-19-vaccine-is-free-so-how-could-you-still-get-a-medical-bill/

[1] Number express quantification of charismatic communication tactics used in message (Jensen et al. 2021; Tur, Harstad, and Antonakis 2021).
