# Supplementary material for: Video-based messages to reduce COVID-19 vaccine hesitancy and nudge vaccination intentions
Source: PLoS One. 2022 Apr 6;17(4):e0265736. doi: 10.1371/journal.pone.0265736 (PMC8985948; doi:10.1371/journal.pone.0265736)
Supplement: S6 Appendix — (PDF) [file pone.0265736.s006.pdf]

S6 Appendix. Estimation Strategy

To estimate the average treatment effect of our messages in figure 1, we estimate the difference in expressed vaccination intentions at T2 as a function of a set of k-1 treatment status indicators (placebo group being the omitted value), and T1 vaccination intention:

$$VacIntent_{T2} = \beta_0 + \beta_1 Treat_{Safety} + \beta_2 Treat_{Norm} + \beta_3 Treat_{Response\ efficacy} + \beta_4 Treat_{Self-efficacy} + \beta_5 VacIntent_{T1} + \boldsymbol{Controls} + e \quad \text{Eq. 1}$$

Or in the pooled case:

$$VacIntent_{T2} = \beta_0 + \beta_1 Treat_{All} + \beta_2 VacIntent_{T1} + \boldsymbol{Controls} + e \quad \text{Eq. 2}$$

Where *e* denotes the error term, and control variables include an indicator for respondent gender, a continuous variable for respondent age, a set of three indicators denoting level of education (high school denoting omitted value), an indicator for whether respondent is white, a set of two indicators denoting political ideology (liberal being the omitted value), an indicator for whether the respondent lives in a rural area.

To estimate the effects of our messages on psychological drivers of vaccine hesitancy in Figure 2, we conduct a similar set of analyses, but swap out vaccination intention for psychological constructs "self-efficacy" (illustrated below) or "response efficacy" (not shown):

$$Self-efficacy_{T2} = \beta_0 + \beta_1 Treat_{Safety} + \beta_2 Treat_{Norm} + \beta_3 Treat_{Response\ efficacy} + \beta_4 Treat_{Self-efficacy} + \beta_5 Self-efficacy_{T1} + \boldsymbol{Controls} + e \quad \text{Eq. 3}$$

Or in the pooled case:

$$Self-efficacy_{T2} = \beta_0 + \beta_1 Treat_{All} + \beta_2 Self-efficacy_{T1} + \boldsymbol{Controls} + e \quad \text{Eq. 4}$$

Instrumental variables regression using two stage least squares is used to estimate the effect of psychological drivers of vaccine hesitancy on vaccination intentions. This estimation procedure involves two stages, in which the exogenous treatment indicators are used in the first state to purge endogeneity from the observed psychological driver, and then subsequently using this exogenous source of variance to predict its relationship with vaccination intention:

$$VacIntent_{T2} = \delta_0 + \delta_1 Self - efficacy + \delta_2 Treat_{Self-efficacy} + \delta_3 VacIntent_{T1} + \boldsymbol{Controls} + v \quad \text{Eq. 5}$$

$$Self - efficacy_{T2} = \beta_0 + \beta_1 Treat_{Self-efficacy} + \beta_2 VacIntent_{T1} + \boldsymbol{Controls} + e \quad \text{Eq. 6}$$

Where v denotes the error term, and control variables include an indicator for respondent gender, a continuous variable for respondent age, a set of three indicators denoting level of education (high school denoting omitted value), an indicator for whether respondent is white, a set of two indicators denoting political ideology (liberal being the omitted value), an indicator for whether the respondent lives in a rural area.
