# Supplementary material for: Video-based messages to reduce COVID-19 vaccine hesitancy and nudge vaccination intentions
Source: PLoS One. 2022 Apr 6;17(4):e0265736. doi: 10.1371/journal.pone.0265736 (PMC8985948; doi:10.1371/journal.pone.0265736)
Supplement: S1 Table — (PDF) [file pone.0265736.s007.pdf]

S1 Table. Summary statistics for sample not fully vaccinated at T2

Variable	Mean	SD	Min	Max
Experimental Group Status (0 "No", 1 "Yes")				
Control Group: Placebo	.253	.435	0	1
Treatment: Safety	.210	.408	0	1
Treatment: Social Norm	.177	.382	0	1
Treatment: Response Efficacy	.181	.386	0	1
Treatment: Self-Efficacy	.179	.384	0	1
Gender (0 "Female", 1 "Male")	.494	.501	0	1
Age	40.65	11.21	19	80
Race/Ethnicity (0 "Non-White", 1 "White")	.774	.442	0	1
Education (0 "No", 1 "Yes")				
High School Degree	.383	.487	0	1
College Degree	.501	.501	0	1
Professional Degree	.107	.310	0	1
Doctorate	.009	.094	0	1
Urban/Rural Living (0 "Urban", 1 "Rural")	.255	.436	0	1
Political Ideology (0 "No", 1 "Yes")				
Conservative	.461	.499	0	1
Moderate	.219	.414	0	1
Liberal	.320	.467	0	1
Trust in Government Institutions (T1)	5.60	2.20	2	10
Vaccination Intention				
T1 (January/February 2021)	4.91	3.89	0	10
T2 (May 2021)	5.03	3.90	0	10
Self-Efficacy				
T1 (January/February 2021)	11.36	3.01	3	15
T2 (May 2021)	13.33	2.55	3	15
Response Efficacy				
T1 (January/February 2021)	10.35	3.75	3	15
T2 (May 2021)	11.04	3.66	3	15
Safety Concerns				
T1 (January/February 2021)	6.60	3.09	2	10
T2 (May 2021)	7.29	2.53	2	10
Protect Others				
T1 (January/February 2021)	6.36	3.29	2	10
T2 (May 2021)	8.85	1.40	2	10

Notes: N =447. N = 228 for variable "trust in government institutions".
