# Supplementary material for: Video-based messages to reduce COVID-19 vaccine hesitancy and nudge vaccination intentions
Source: PLoS One. 2022 Apr 6;17(4):e0265736. doi: 10.1371/journal.pone.0265736 (PMC8985948; doi:10.1371/journal.pone.0265736)
Supplement: S2 Table — (PDF) [file pone.0265736.s008.pdf]

S2 Table. Summary statistics for full sample

Variable	Mean	SD	Min	Max
Experimental Group Status (0 "No", 1 "Yes")				
Control Group: Placebo	.236	.425	0	1
Treatment: Safety	.216	.412	0	1
Treatment: Social Norm	.170	.376	0	1
Treatment: Response Efficacy	.186	.390	0	1
Treatment: Self-Efficacy	.192	.394	0	1
Gender (0 "Female", 1 "Male")	.511	.500	0	1
Age	42.31	12.01	19	84
Race/Ethnicity (0 "Non-White", 1 "White")	.763	.426	0	1
Education (0 "No", 1 "Yes")				
High School Degree	.313	.464	0	1
College Degree	.544	.498	0	1
Professional Degree	.135	.342	0	1
Doctorate	.007	.084	0	1
Urban/Rural Living (0 "Urban", 1 "Rural")	.235	.424	0	1
Political Ideology (0 "No", 1 "Yes")				
Conservative	.357	.479	0	1
Moderate	.216	.412	0	1
Liberal	.427	.495	0	1
Trust in Government Institutions (T1)	6.26	2.12	2	10
Vaccination Intention				
T1 (January/February 2021)	6.86	3.71	0	10
T2 (May 2021)	7.30	3.75	0	10
Vaccination Uptake - T2 (0 "Not Vaccinated", 1 "Vaccinated")	.572	.495	0	1
CDC Vaccination Card - T2 (0 "Not Shared", 1 "Shared")	.221	.415	0	1
Self-Efficacy				
T1 (January/February 2021)	12.34	2.77	3	15
T2 (May 2021)	14.02	2.07	3	15
Response Efficacy				
T1 (January/February 2021)	11.66	3.39	3	15
T2 (May 2021)	12.43	3.22	3	15
Safety Concerns				
T1 (January/February 2021)	5.24	3.14	2	10
T2 (May 2021)	5.68	2.83	2	10
Protect Others				
T1 (January/February 2021)	7.29	3.18	2	10
T2 (May 2021)	9.22	1.24	2	10

Notes: N =843. N = 415 for variable "trust in government institutions".
