# Supplementary material for: Video-based messages to reduce COVID-19 vaccine hesitancy and nudge vaccination intentions
Source: PLoS One. 2022 Apr 6;17(4):e0265736. doi: 10.1371/journal.pone.0265736 (PMC8985948; doi:10.1371/journal.pone.0265736)
Supplement: S3 Table — (PDF) [file pone.0265736.s009.pdf]

S3 Table. Correlation matrix for sample of not fully vaccinated individuals

	(1)	(2)	(3)	(4)	(5)	(6)	(7)	(8)	(9)
1 Group: Placebo	.								
2 Group: Treatment - Safety	-0.30***	.							
3 Group: Treatment - Social Norm	-0.27***	-0.24***	.						
4 Group: Treatment - Response Efficacy	-0.27***	-0.24***	-0.22***	.					
5 Group: Treatment - Self-Efficacy	-0.27***	-0.24***	-0.22***	-0.22***	.				
6 Vaccination Intention (T1)	0.00	0.02	0.01	-0.01	-0.02	.			
7 Vaccination Intention (T2)	0.10*	-0.01	-0.00	-0.06	-0.05	0.77***	.		
8 Vaccination Uptake (T2)	-0.01	0.04	-0.00	0.05	-0.08	0.53***	0.38***	.	
9 CDC Vaccination Card (T2)	0.03	0.02	0.03	-0.04	-0.04	0.33***	0.24***	0.56***	.
10 Gender (Male)	-0.06	-0.04	0.02	-0.01	0.10*	0.11*	0.12*	0.05	0.05
11 Age	0.02	0.01	-0.00	-0.06	0.03	-0.08	-0.10*	-0.05	0.05
12 Education: High School Degree	-0.02	0.00	0.05	0.05	-0.07	-0.16***	-0.13**	-0.17***	-0.12*
13 Education: College Degree	0.01	-0.02	-0.02	-0.03	0.06	0.16***	0.11*	0.16***	0.06
14 Education: Professional Degree	-0.00	0.03	-0.03	-0.03	0.03	-0.02	-0.00	-0.00	0.07
15 Education: Doctorate	0.05	0.01	-0.04	0.02	-0.04	0.06	0.08	0.07	0.06
16 Race/Ethnicity (White)	0.00	0.01	0.05	0.01	-0.08	-0.10*	-0.13**	-0.05	-0.06
17 Liberal	-0.05	-0.01	0.05	0.05	-0.03	0.33***	0.36***	0.19***	0.15**
18 Moderate	-0.10*	-0.03	0.01	0.05	0.09	-0.01	-0.06	0.03	-0.00
19 Conservative	0.12**	0.04	-0.05	-0.09	-0.05	-0.30***	-0.28***	-0.20***	-0.14**
20 Rural	-0.07	0.00	0.04	0.11*	-0.07	-0.11*	-0.13**	-0.12*	-0.11*
21 Self-Efficacy (T1)	0.04	-0.01	0.02	-0.05	-0.01	0.25***	0.31***	0.15**	0.15**
22 Self-Efficacy (T2)	-0.09	0.06	-0.00	0.01	0.02	0.27***	0.21***	0.22***	0.16**
23 Response Efficacy (T1)	0.05	-0.00	0.01	-0.07	0.00	0.61***	0.69***	0.28***	0.23***
24 Response Efficacy (T2)	-0.03	-0.01	0.08	0.04	-0.06	0.69***	0.62***	0.30***	0.21***
25 Safety Concern (T1)	-0.02	0.00	0.01	0.02	-0.01	-0.49***	-0.60***	-0.21***	-0.13**
26 Safety Concern (T2)	-0.03	0.04	-0.05	-0.01	0.04	-0.71***	-0.66***	-0.40***	-0.29***
27 Desire to Protect (T1)	0.04	-0.02	0.03	-0.09	0.03	0.53***	0.63***	0.28***	0.21***
28 Desire to Protect (T2)	-0.06	0.05	-0.02	0.01	0.03	0.29***	0.27***	0.16***	0.16***

N = 447. *** $p<0.001$, ** $p<0.01$, * $p<0.5$.

S3 Table. Correlation matrix for sample of not fully vaccinated individuals (cont.)

	(10)	(11)	(12)	(13)	(14)	(15)	(16)	(17)	(18)
10 Gender (Male)	.								
11 Age	-0.13**	.							
12 Education: High School Degree	-0.01	-0.04	.						
13 Education: College Degree	0.05	-0.01	-0.79***	.					
14 Education: Professional Degree	-0.08	0.07	-0.27***	-0.35***	.				
15 Education: Doctorate	0.05	0.02	-0.07	-0.10*	-0.03	.			
16 Race/Ethnicity (White)	-0.06	0.05	0.07	-0.03	-0.05	0.00	.		
17 Liberal	0.02	-0.19***	0.02	0.00	-0.05	0.04	-0.21***	.	
18 Moderate	-0.04	0.10*	0.08	-0.11*	0.06	-0.05	-0.02	-0.36***	.
19 Conservative	0.01	0.10*	-0.09	0.09	-0.00	0.01	0.21***	-0.63***	-0.49***
20 Rural	-0.11*	-0.03	0.20***	-0.16***	-0.05	-0.06	0.21***	-0.10*	0.01
21 Self-Efficacy (T1)	0.04	0.03	-0.10*	0.08	0.02	0.03	-0.05	0.06	-0.09
22 Self-Efficacy (T2)	-0.01	0.01	-0.06	0.01	0.06	0.03	-0.02	0.02	-0.01
23 Response Efficacy (T1)	0.11*	-0.04	-0.10*	0.06	0.05	0.07	-0.15**	0.31***	-0.04
24 Response Efficacy (T2)	0.09	-0.07	-0.08	0.09	-0.03	0.06	-0.06	0.26***	0.00
25 Safety Concern (T1)	-0.21***	0.22***	0.11*	-0.14**	0.07	-0.06	0.07	-0.24***	-0.01
26 Safety Concern (T2)	-0.25***	0.18***	0.17***	-0.18***	0.04	-0.07	0.07	-0.30***	0.04
27 Desire to Protect (T1)	0.01	-0.02	-0.12*	0.09	0.03	0.04	-0.07	0.21***	-0.08
28 Desire to Protect (T2)	-0.09	0.06	-0.09	0.06	0.04	0.01	-0.15**	0.10*	0.00

N = 447. *** p<0.001, ** p<0.01, * p<0.5.

S3 Table. Correlation matrix for sample of not fully vaccinated individuals (cont.)

	(19)	(20)	(21)	(22)	(23)	(24)	(25)	(26)	(27)	(28)
19 Conservative	.									
20 Rural	0.09	.								
21 Self-Efficacy (T1)	0.02	-0.00	0.73							
22 Self-Efficacy (T2)	-0.01	-0.01	0.49***	0.84						
23 Response Efficacy (T1)	-0.25***	-0.17***	0.28***	0.23***	0.92					
24 Response Efficacy (T2)	-0.24***	-0.12**	0.24***	0.29***	0.72***	0.92				
25 Safety Concern (T1)	0.24***	0.05	-0.14**	-0.09*	-0.47***	-0.42***	0.69			
26 Safety Concern (T2)	0.24***	0.12*	-0.22***	-0.19***	-0.53***	-0.60***	0.60***	0.61		
27 Desire to Protect (T1)	-0.13**	-0.16***	0.22***	0.19***	0.54***	0.49***	-0.27***	-0.40***	0.50	
28 Desire to Protect (T2)	-0.09*	0.04	0.30***	0.39***	0.26***	0.27***	-0.06	-0.17***	0.33***	0.72

N = 447. *** p<0.001, ** p<0.01, * p<0.5. In columns 21-24, the diagonal entry denotes the alpha reliability coefficient of the three items used to generate the self-efficacy and response efficacy scales, respectively. The diagonal entry for columns 25-28 denotes the pairwise correlation coefficient for the two items used to generate the scales for safety concerns and desire to protect, respectively. Variable "trust in government" is omitted due to lower number of respondents (n = 228). Pairwise correlations between this variable and other study variables can be generated using the open data and code materials.
