# Supplementary material for: Video-based messages to reduce COVID-19 vaccine hesitancy and nudge vaccination intentions
Source: PLoS One. 2022 Apr 6;17(4):e0265736. doi: 10.1371/journal.pone.0265736 (PMC8985948; doi:10.1371/journal.pone.0265736)
Supplement: S4 Table — Respondents failing attention check included. (PDF) [file pone.0265736.s010.pdf]

S4 Table. Increase in vaccination intentions (T2) after watching treatment videos. Respondents failing attention check included.

	Model 1	Model 2	Model 3	Model 4
Experimental Group *(Ref. = Placebo)*				
Treatments (Pooled)	0.70***	0.56**		
	(2.90)	(2.24)		
Treatment: Safety			0.78**	0.68**
			(2.34)	(2.05)
Treatment: Social Norm			0.62*	0.49
			(1.93)	(1.52)
Treatment: Response Efficacy			0.73**	0.56*
			(2.22)	(1.66)
Treatment: Self-Efficacy			0.65*	0.46
			(1.76)	(1.19)
Vaccination Intention (T1)	0.78***	0.74***	0.78***	0.73***
	(29.09)	(21.42)	(28.88)	(21.15)
Man *(Ref. = Woman)*		0.16		0.17
		(0.66)		(0.71)
Age		0.00		0.00
		(0.18)		(0.19)
Education *(Ref. = High School)*				
College Degree		0.74***		0.75***
		(2.89)		(2.91)
Professional Degree		0.20		0.20
		(0.55)		(0.54)
Doctorate		0.52		0.49
		(0.57)		(0.55)
Race/Ethnicity *(Ref. = Non-White)*		0.25		0.24
		(0.98)		(0.95)
Political Ideology *(Ref. = Liberal)*				
Moderate		-0.25		-0.24
		(-0.76)		(-0.73)
Conservative		-0.86***		-0.87***
		(-2.94)		(-2.96)
Rural *(Ref. = Urban)*		0.04		0.04
		(0.15)		(0.15)
Constant	0.65***	0.68	0.65***	0.68
	(2.68)	(1.21)	(2.67)	(1.20)
Observations	467	464	467	464
R-squared	0.61	0.62	0.61	0.62

Notes: *** p<0.01, ** p<0.05, * p<0.1. Robust t-statistics in parentheses. ATE estimated using OLS regressions, showing unstandardized regression coefficient estimates. Two-sided tests.
