# Supplementary material for: Video-based messages to reduce COVID-19 vaccine hesitancy and nudge vaccination intentions
Source: PLoS One. 2022 Apr 6;17(4):e0265736. doi: 10.1371/journal.pone.0265736 (PMC8985948; doi:10.1371/journal.pone.0265736)
Supplement: S5 Table — OLS regressions. (PDF) [file pone.0265736.s011.pdf]

S5 Table. Increase in vaccination intentions (T2) after watching treatment videos. OLS regressions.

	Model 1	Model 2	Model 3	Model 4
Experimental Group *(Ref. = Placebo)*				
Treatments (Pooled)	0.69***	0.55**		
	(2.79)	(2.18)		
Treatment: Safety			0.73**	0.67*
			(2.10)	(1.95)
Treatment: Social Norm			0.59*	0.45
			(1.78)	(1.35)
Treatment: Response Efficacy			0.78**	0.61*
			(2.33)	(1.79)
Treatment: Self-Efficacy			0.65*	0.46
			(1.74)	(1.15)
Vaccination Intention (T1)	0.78***	0.74***	0.78***	0.74***
	(28.02)	(21.10)	(27.78)	(20.83)
Man *(Ref. = Woman)*		0.15		0.17
		(0.62)		(0.67)
Age		0.00		0.00
		(0.11)		(0.13)
Education *(Ref. = High School)*				
College Degree		0.71***		0.72***
		(2.72)		(2.73)
Professional Degree		0.17		0.17
		(0.45)		(0.45)
Doctorate		0.49		0.45
		(0.55)		(0.50)
Race/Ethnicity *(Ref. = Non-White)*		0.22		0.22
		(0.84)		(0.82)
Political Ideology *(Ref. = Liberal)*				
Moderate		-0.12		-0.11
		(-0.36)		(-0.33)
Conservative		-0.81***		-0.81***
		(-2.70)		(-2.71)
Rural *(Ref. = Urban)*		0.01		0.01
		(0.05)		(0.03)
Constant	0.69***	0.73	0.69***	0.72
	(2.77)	(1.28)	(2.74)	(1.25)
Observations (Unique Individuals)	447	447	447	447
R-squared	0.60	0.61	0.60	0.61

Notes: *** $p<0.01$, ** $p<0.05$, * $p<0.1$. Robust t-statistics in parentheses. ATE estimated using OLS regressions, showing unstandardized regression coefficient estimates. Two-sided tests.
