# Supplementary material for: Video-based messages to reduce COVID-19 vaccine hesitancy and nudge vaccination intentions
Source: PLoS One. 2022 Apr 6;17(4):e0265736. doi: 10.1371/journal.pone.0265736 (PMC8985948; doi:10.1371/journal.pone.0265736)
Supplement: S6 Table — OLS regressions. (PDF) [file pone.0265736.s012.pdf]

S6 Table. T2 vaccination status predicted by T1 vaccination intention and controls. OLS regressions.

	Self-Reported Vaccination Status (T2)		Verified Vaccination Status (T2)	
	Model 1	Model 2	Model 3	Model 4
Vaccination Intention (T1)	0.083***	0.066***	0.033***	0.018***
	(29.45)	(9.95)	(11.35)	(2.85)
Man *(Ref. = Woman)*		-0.008		0.016
		(-0.29)		(0.57)
Age		0.003***		0.002*
		(2.65)		(1.66)
Education *(Ref. = High School)*				
College Degree		0.101***		0.041
		(3.10)		(1.27)
Professional Degree		0.094**		0.000
		(2.29)		(0.01)
Doctorate		0.111		0.089
		(0.48)		(0.53)
Race/Ethnicity *(Ref. = Non-White)*		0.069*		-0.070*
		(1.92)		(-1.94)
Political Ideology *(Ref. = Liberal)*				
Moderate		-0.028		-0.038
		(-0.76)		(-0.94)
Conservative		-0.122***		-0.069*
		(-3.48)		(-1.82)
Rural *(Ref. = Urban)*		-0.030		0.002
		(-0.86)		(0.05)
Response Efficacy (T1)		-0.000		0.003
		(-0.04)		(0.64)
Self-Efficacy (T1)		0.022***		0.011**
		(4.03)		(2.39)
Safety Concern (T1)		-0.009		-0.007
		(-1.42)		(-1.10)
Desire to Protect (T1)		-0.007		0.003
		(-1.27)		(0.51)
Constant	0.001	-0.532***	-0.004	-0.064
	(0.05)	(-2.99)	(-0.25)	(-0.36)
State Fixed Effects	NO	YES	NO	YES
Observations (Unique Individuals)	843	843	843	843
R-squared	0.39	0.47	0.09	0.13

Robust t-statistics in parentheses, *** p<0.01, ** p<0.05, * p<0.1.
