# Supplementary material for: Video-based messages to reduce COVID-19 vaccine hesitancy and nudge vaccination intentions
Source: PLoS One. 2022 Apr 6;17(4):e0265736. doi: 10.1371/journal.pone.0265736 (PMC8985948; doi:10.1371/journal.pone.0265736)
Supplement: S7 Table — OLS regressions. (PDF) [file pone.0265736.s013.pdf]

S7 Table. Increase in self-efficacy (T2) after watching treatment videos. OLS regressions.

	Model 1	Model 2	Model 3	Model 4
Experimental Group *(Ref. = Placebo)*				
Treatments (Pooled)	0.64***	0.66***		
	(2.76)	(2.77)		
Treatment: Safety			0.80***	0.80***
			(2.71)	(2.69)
Treatment: Social Norm			0.41	0.45
			(1.30)	(1.41)
Treatment: Response Efficacy			0.70**	0.72**
			(2.20)	(2.17)
Treatment: Self-Efficacy			0.62*	0.64*
			(1.85)	(1.88)
Self-Efficacy (T1)	0.42***	0.42***	0.42***	0.42***
	(8.57)	(8.50)	(8.56)	(8.50)
Man *(Ref. = Woman)*		-0.18		-0.17
		(-0.81)		(-0.76)
Age		-0.00		-0.00
		(-0.46)		(-0.44)
Education *(Ref. = High School)*				
College Degree		-0.05		-0.05
		(-0.20)		(-0.21)
Professional Degree		0.34		0.33
		(1.05)		(1.03)
Doctorate		0.78		0.72
		(1.23)		(1.11)
Race/Ethnicity *(Ref. = Non-White)*		0.05		0.06
		(0.20)		(0.25)
Political Ideology *(Ref. = Liberal)*				
Moderate		0.17		0.17
		(0.60)		(0.60)
Conservative		0.07		0.06
		(0.29)		(0.24)
Rural *(Ref. = Urban)*		-0.14		-0.14
		(-0.58)		(-0.58)
Constant	8.08***	8.21***	8.06***	8.18***
	(12.44)	(10.86)	(12.36)	(10.74)
Observations (Unique Individuals)	447	447	447	447
R-squared	0.26	0.26	0.26	0.26

Notes: *** p<0.01, ** p<0.05, * p<0.1. Robust t-statistics in parentheses. ATE estimated using OLS regressions, showing unstandardized regression coefficient estimates. Two-sided tests.
