# Supplementary material for: Video-based messages to reduce COVID-19 vaccine hesitancy and nudge vaccination intentions
Source: PLoS One. 2022 Apr 6;17(4):e0265736. doi: 10.1371/journal.pone.0265736 (PMC8985948; doi:10.1371/journal.pone.0265736)
Supplement: S8 Table — OLS regressions. (PDF) [file pone.0265736.s014.pdf]

S8 Table. Increase in response efficacy (T2) after watching treatment videos. OLS regressions.

	Model 1	Model 2	Model 3	Model 4
Experimental Group *(Ref. = Placebo)*				
Treatments (Pooled)	0.56**	0.50*		
	(2.00)	(1.77)		
Treatment: Safety			0.34	0.33
			(0.93)	(0.93)
Treatment: Social Norm			0.94***	0.86**
			(2.71)	(2.38)
Treatment: Response Efficacy			1.10***	1.01***
			(3.08)	(2.86)
Treatment: Self-Efficacy			-0.09	-0.14
			(-0.22)	(-0.35)
Response Efficacy (T1)	0.70***	0.69***	0.71***	0.69***
	(20.51)	(18.19)	(20.77)	(18.43)
Man *(Ref. = Woman)*		0.03		0.06
		(0.10)		(0.25)
Age		-0.01		-0.01
		(-0.99)		(-0.85)
Education *(Ref. = High School)*				
College Degree		0.29		0.33
		(1.13)		(1.30)
Professional Degree		-0.53		-0.46
		(-1.22)		(-1.08)
Doctorate		0.88		0.80
		(0.92)		(0.80)
Race/Ethnicity *(Ref. = Non-White)*		0.49*		0.44
		(1.78)		(1.61)
Political Ideology *(Ref. = Liberal)*				
Moderate		0.06		0.13
		(0.20)		(0.42)
Conservative		-0.47*		-0.40
		(-1.66)		(-1.42)
Rural *(Ref. = Urban)*		-0.06		-0.15
		(-0.22)		(-0.50)
Constant	3.35***	3.69***	3.31***	3.57***
	(7.00)	(4.98)	(6.95)	(4.86)
Observations (Unique Individuals)	447	447	447	447
R-squared	0.52	0.53	0.53	0.54

Notes: *** p<0.01, ** p<0.05, * p<0.1. Robust t-statistics in parentheses. ATE estimated using OLS regressions, showing unstandardized regression coefficient estimates. Two-sided tests.
