# Supplementary material for: Video-based messages to reduce COVID-19 vaccine hesitancy and nudge vaccination intentions
Source: PLoS One. 2022 Apr 6;17(4):e0265736. doi: 10.1371/journal.pone.0265736 (PMC8985948; doi:10.1371/journal.pone.0265736)
Supplement: S9 Table — (PDF) [file pone.0265736.s015.pdf]

S9 Table. Response efficacy and self-efficacy as psychological mechanisms driving effect of video messages on vaccination intentions.

Stage:	First	Second	First	Second
Dependent Variable (T2):	Self-Efficacy	Vaccine Intention	Response Efficacy	Vaccine Intention
Experimental Messages *(Ref. = Placebo)*				
Treatment: Response Efficacy			1.16***	
			(2.91)	
Treatment: Self-Efficacy	1.00**			
	(2.51)			
Self-Efficacy (T2)		0.73*		
		(1.69)		
Response Efficacy (T2)				0.57*
				(1.83)
Vaccination Intention (T1)	0.17**	0.62***	0.66***	0.44**
	(2.54)	(7.20)	(11.30)	(2.31)
Man *(Ref. = Woman)*	-0.83**	-0.48	0.12	-0.35
	(-2.10)	(-0.10)	(0.28)	(-0.98)
Age	-0.01	0.01	-0.01	-0.01
	(-0.63)	(0.36)	(-0.47)	(-0.55)
Education *(Ref. = High School)*				
College Degree	-0.30	0.98**	-0.08	0.89**
	(-0.65)	(2.01)	(-0.17)	(2.47)
Professional Degree	0.68	-0.22	-0.43	0.40
	(1.52)	(-0.32)	(-0.51)	(0.65)
Doctorate	1.48***	-0.22	0.32	-0.07
	(2.68)	(-0.21)	(0.22)	(-0.15)
Race/Ethnicity *(Ref. = Non-White)*	-0.12	0.47	0.48	0.06
	(-0.30)	(1.03)	(1.09)	(0.16)
Political Ideology *(Ref. = Liberal)*				
Moderate	-0.48	-0.31	0.68	0.38
	(-0.88)	(-0.55)	(1.21)	(0.74)
Conservative	0.54	-0.94*	-0.34	-0.22
	(1.27)	(-1.73)	(-0.70)	(-0.51)
Rural *(Ref. = Urban)*	0.08	-0.21	-0.42	0.43
	(0.17)	(-0.48)	(-0.84)	(1.21)
Constant	12.75***	-8.34	7.38***	-3.58
	(1.11)	(-1.44)	(7.71)	(-1.30)
Observations	193	193	194	194
R-squared	0.09	0.48	0.48	0.69

Notes: *** $p<0.01$, ** $p<0.05$, * $p<0.1$. Two-stage least-squares instrumental variable regression using video treatments as experimentally randomized instrumental variables. Regression coefficients report first and second stage results. Heteroscedastic robust t-statistic in parentheses. Both regression models are exactly identified.
