# Supplementary material for: Video-based messages to reduce COVID-19 vaccine hesitancy and nudge vaccination intentions
Source: PLoS One. 2022 Apr 6;17(4):e0265736. doi: 10.1371/journal.pone.0265736 (PMC8985948; doi:10.1371/journal.pone.0265736)
Supplement: S10 Table — (PDF) [file pone.0265736.s016.pdf]

S10 Table. Heterogenous treatment effect on vaccination intentions by political ideology

Experimental Group *(Ref. = Placebo)*	
Treatments (Pooled)	0.27
	(0.63)
Political Ideology (PI)	-0.56**
	(-2.31)
Treatments * PI	0.23
	(0.77)
Vaccination Intention (T1)	0.71***
	(17.54)
Man *(Ref. = Woman)*	0.18
	(0.66)
Age	0.00
	(0.36)
Education *(Ref. = High School)*	
College Degree	0.67**
	(2.35)
Professional Degree	0.10
	(0.27)
Doctorate	0.38
	(0.32)
Race/Ethnicity *(Ref. = Non-White)*	0.22
	(0.79)
Rural *(Ref. = Urban)*	0.05
	(0.15)
Constant	1.11
	(1.56)
Observations	447
R-squared	0.65

Notes: *** p<0.01, ** p<0.05, * p<0.1. Robust t-statistics in parentheses with k-1 state dummies. LATE estimated using OLS regressions, showing unstandardized regression coefficient estimates. Two-sided tests. PI is treated as continuous with three categories: 1 “liberal”, 2 “moderate”, and 3 “conservative”.
