# Supplementary material for: Video-based messages to reduce COVID-19 vaccine hesitancy and nudge vaccination intentions
Source: PLoS One. 2022 Apr 6;17(4):e0265736. doi: 10.1371/journal.pone.0265736 (PMC8985948; doi:10.1371/journal.pone.0265736)
Supplement: S11 Table — (PDF) [file pone.0265736.s017.pdf]

S11 Table. Heterogenous treatment effect on vaccination intentions by trust in government institutions

Experimental Group *(Ref. = Placebo)*	
Treatments (Pooled)	2.13*
	(1.92)
Trust in Government Institutions	0.36**
	(2.01)
Treatments * Trust in Government Institutions	-0.31
	(-1.63)
Vaccination Intention (T1)	0.63***
	(9.43)
Man *(Ref. = Woman)*	0.30
	(0.70)
Age	0.01
	(0.62)
Education *(Ref. = High School)*	
College Degree	0.37
	(0.81)
Professional Degree	-0.08
	(-0.11)
Doctorate	0.76
	(0.87)
Race/Ethnicity *(Ref. = Non-White)*	0.20
	(0.46)
Political Ideology *(Ref. = Liberal)*	
Moderate	-0.64
	(-1.32)
Conservative	-1.51***
	(-3.09)
Rural *(Ref. = Urban)*	0.18
	(0.47)
Constant	-0.82
	(-0.55)
Observations	228
R-squared	0.69

Notes: *** $p<0.01$, ** $p<0.05$, * $p<0.1$. Robust t-statistics in parentheses with k-1 state dummies. LATE estimated using OLS regressions, showing unstandardized regression coefficient estimates. Two-sided tests. Trust in government institutions is a continuous scale ranging from 2 to 10 comprised of two 5-point Likert scaled items: (1) how much do you trust the U.S. Government to provide accurate information on COVID-19? and (2) how much do you trust the Coronavirus Taskforce to provide accurate information on COVID-19?
